# From Petri Dish to Patient: Bioavailability Estimation and Mechanism of Action for Antimicrobial and Immunomodulatory Natural Products

**DOI:** 10.3389/fmicb.2019.02470

**Published:** 2019-10-31

**Authors:** Nicholas John Sadgrove, Graham Lloyd Jones

**Affiliations:** ^1^Pharmaceuticals and Nutraceuticals (PAN) Group, School of Science and Technology, University of New England, Armidale, NSW, Australia; ^2^Jodrell Science Laboratory, Royal Botanic Gardens, Kew, Richmond, United Kingdom

**Keywords:** polar head space, rotatable bonds, pharmacokinetics, pharmacodynamics, transdermal penetration

## Abstract

The new era of multidrug resistance of pathogens against frontline antibiotics has compromised the immense therapeutic gains of the ‘golden age,’ stimulating a resurgence in antimicrobial research focused on antimicrobial and immunomodulatory components of botanical, fungal or microbial origin. While much valuable information has been amassed on the potency of crude extracts and, indeed, purified compounds there are too many reports that uncritically extrapolate observed *in vitro* activity to presumed ingestive and/or topical therapeutic value, particularly in the discipline of ethnopharmacology. Thus, natural product researchers would benefit from a basic pharmacokinetic and pharmacodynamic understanding. Furthermore, therapeutic success of complex mixtures or single components derived therefrom is not always proportionate to their MIC values, since immunomodulation can be the dominant mechanism of action. Researchers often fail to acknowledge this, particularly when ‘null’ activity is observed. In this review we introduce the most up to date theories of oral and topical bioavailability including the metabolic processes affecting xenobiotic biotransformation before and after drugs reach the site of their action in the body. We briefly examine the common methodologies employed in antimicrobial, immunomodulatory and pharmacokinetic research. Importantly, we emphasize the contribution of synergies and/or antagonisms in complex mixtures as they affect absorptive processes in the body and sometimes potentiate activity. Strictly in the context of natural product research, it is important to acknowledge the potential for chemotypic variation within important medicinal plants. Furthermore, polar head space and rotatable bonds give *a priori* indications of the likelihood of bioavailability of active metabolites. Considering this and other relatively simple chemical insights, we hope to provide the basis for a more rigorous scientific assessment, enabling researchers to predict the likelihood that observed *in vitro* anti-infective activity will translate to *in vivo* outcomes in a therapeutic context. We give worked examples of tentative pharmacokinetic assessment of some well-known medicinal plants.

## Introduction

The pharmacotherapeutic value of antimicrobial and immunomodulatory (anti-infective) drugs critically depends on the orchestration of properties influencing pharmacokinetics and pharmacodynamics. In the former, the discipline of pharmacokinetics was born out of the need to monitor and maintain optimal physiological concentration of a drug to achieve a positive therapeutic outcome. Optimal concentration is above an ‘active’ threshold but below contraindicated (and possibly toxic) levels. In clinical practice, to achieve optimal concentration, factors under consideration include efficiency of absorption, drug half-life and hence, dose and intervals of drug administration. In a broader sense, characteristics influencing pharmacokinetic fate of a specific drug critically depend on its chemical functional groups, which are the basis for *a priori* insight into the possibility of absorption or transdermal penetration.

However, pharmacodynamics is a more preliminary step in that the mechanism of the drug is fully or tentatively explained and the therapeutic and/or toxicity thresholds are established. One of the bigger challenges in pharmacodynamics is the translation to the pharmacokinetic context, from *in vitro* models to *in vivo* environments where several physiological processes may compromise the presumed positive therapeutic outcomes. The most important of these challenges include absorption and biotransformation of ingested therapies occurring in the liver and by the gut microbiota. Such challenges are commonly neglected in ethnopharmacological or natural product research, particularly those involving crude extracts as commonly administered in herbal medicine.

After the turn of the century most of the research concerning anti-infectives has focused on natural plant, marine or endophyte extracts. Since this research usually starts with a bioactivity guided fractionation of a crude extract, structural elucidation studies commence at a relatively late stage. Ideally, the preliminary steps taken before measuring biological activity should involve tentative interpretation in the context of pharmacokinetics by closer examination of polar functional groups known to influence absorption and the number of rotatable bonds. This is critical when therapies are ingested and expected to act non-locally (not in the digestive tract) and therefore require sufficiently high systemic concentration. Understandably, since most investigations measure activity first and compound structure second, with pharmacokinetic interpretation as a final step, there are a plethora of published studies reporting successful *in vitro* outcomes which are naively and often uncritically extrapolated to presumed *in vivo* therapeutic value.

Fortunately, by remembering only a few generic guidelines a greater understanding of pharmacokinetics can be acquired, empowering the researcher to more critically assess *in vitro* outcomes for potential *in vivo* reproducibility. Thus, the current review highlights the most common problems with the presentation of *in vitro* outcomes and provides insight into how data could be interpreted to provide more relevant conclusions on therapeutic potential.

## The ‘Dark Age’ of Antibiotics

The ‘dark age’ of antibiotics is a time of resistance development against the antibiotics discovered in the ‘golden era’ (Lyddiard et al., 2016). Ironically, even before the ‘Waksman platform,’ which led to the discovery of most of the antibiotics in use today, we were warned that this time would come. On receipt of his 1945 Nobel prize for the discovery of penicillin, Alexander Fleming made the prescient observation that the ‘*thoughtless person playing with penicillin treatment is morally responsible for the death of the man who succumbs to infection with the penicillin-resistant organism*’ (Cheesman et al., 2017).

It could be argued that the modern techniques of molecular docking and rational drug design have demonstrated little success by comparison with the much less sophisticated screening methods employed during the ‘golden era’ of antibiotic discovery and this gives impetus to calls for a new iteration of natural product screening in the search for new efficacious drugs and novel drug scaffolds (Lyddiard et al., 2016). Yet another lesson we could learn from the ‘dark age,’ and Fleming’s grim yet accurate prediction, is to direct research efforts toward development of combination therapy drugs, by contrast with the monotherapy drug approach that ushered in the resistance paradigm (Cock, 2018).

Most of the antimicrobial compounds identified as secondary metabolites from natural products have a low degree of specificity in their mechanisms of action (MOA) and yet the most successful antibiotics have a high degree of specificity. This prompts the question of whether there is a correlation between degree of specificity and potency of drugs that are safe in human use. If this is indeed true, then the trade-off may be that with higher specificity and potency comes greater probability of resistance development. Adjuvants can, in some cases, disrupt resistance mechanisms (Cock, 2018), but combination therapies that target two or more sites provide arguably the best strategy for preventing further resistance development. Thus, this new paradigm of dual-therapy drugs opens a potential niche for the common non-specific antimicrobials found in natural product research that could be used to complement the conventional antibiotics that are losing potency in the unrelenting march of microbial resistance.

## Pharmacodynamics of Drugs

### Mechanism of Action (MOA) and Structure-Activity Relationships (SAR)

Van Vuuren and Holl (2017) suggested that the criteria for describing the levels of antimicrobial activity in natural products be specific for types of extract, using the terms ‘moderate,’ ‘strong,’ ‘very strong,’ and ‘noteworthy’. In the case of crude extracts from medicinal plants, noteworthy activity is ascribed for activity ≤160 μg/ml, for essential oils it is ≤1000 μg/ml and for pure compounds it is ≤16 μg/ml.

In antimicrobial research a pronounced distinction can be made between susceptibility of Gram-negative and Gram-positive bacteria, where Gram-negative organisms tend to be less susceptible on average. This is due to the presence of an outer membrane and hydrophilic periplasmic space in Gram-negative bacteria, which influences penetration and the fate of antibiotics. Thus, there are many antibiotics that have specificity for the Gram-positive organisms. For example, vancomycin is too large to cross the outer cell membrane of Gram-negative bacteria and thus has little to no activity against them. Furthermore, in the context of penicillins, Gram-negative bacteria have a privileged site for the accumulation of β-lactamases, with increased expression in the presence of β-lactam antibiotics (Zeng and Lin, 2013); a resistance mechanism that substantially reduces penicillin efficacy. However, antibiotics with broad spectrum activity have activity across both Gram-types. All such antibiotics have α-hydrophilic groups which aid passage across the lipophobic periplasmic space of Gram-negative bacteria (Patrick, 2013).

For the most successful antibiotics currently in use, five main categories of mode of action are known, which are: (1) Inhibition of enzyme activity (antimetabolites), (2) Disruption of cell wall synthesis, (3) Plasma membrane interference, (4) Prevention of protein synthesis at ribosomes, and (5) Inhibition of DNA transcription and replication. The main types of drugs used in the pharmaceutical industry and their mechanisms are listed in Tables 1, 2.

**TABLE 1:** The penicillins; their chemical groups and subtle differences in modes of action (MOA) against bacterial cell wall biosynthesis (Patrick, 2013).

**β-lactam penicillins, COR-substituted 6-aminopenicillanic acid (acyl derivatives of 6-APA)**							

**Sub-group**	**Benzylpenicillin**	**Aminopenicillins**	**Carboxypenicillins**	**Ureidopenicillins**	**Acid-resistant**	**Penicillinase-resistant**	**Penicillin prodrug**
Sub-structure	6-benzyl substituted	6-amino substituted	α-carboxy substituted	α-urea substituted	6-electron withdrawal substituted	6-steric shield substituted	Ampicillin esters of carboxyl moiety

**MOA**	**Cell wall, inhibition of transpeptidase enzyme**						

Sub-MOA	Lipophilic, but unstable in stomach acid	Broad spectrum, higher hydrophilicity, crosses Gram-negative cell wall			Stable in stomach acid	Blocks β-lactamase	Ampicillin has poor absorption, esters aid absorption and are hydrolyzed in phase-1 metabolism

**Inhibition**	**Bactericidal**						

Examples	Penicillin G	Ampicillin, Amoxicillin, Penicillin N, Penicillin T	Carbenicillin, carfecillin (prodrug), ticarcillin	Azlocillin, mezlocillin, piperacillin	Penicillin V, ampicillin, amoxicillin	Methicillin, nafcillin, temocillin, oxacillin, cloxacillin, flucloxacillin, dicloxacillin	Pivampicillin, talampicillin, bacampicillin

**TABLE 2:** Other frontline antibiotics (Patrick, 2013).

**Group**	**Glycopeptides**	**Aminoglycosides**	**Tetracyclines**	**Macrolides**	**Chloramphenicol**	**Lincosamides**	**Streptogramins**
Sub-structure	Polyphenolic glycopeptides	Carbohydrate, basic amine groups	Tetracyclic, two enols, one amide	O-glycosylated lactone rings	Dichloroacetamide, nitrophenyl	Thiosugar amine	Lactone macrocycles
MOA	Bind to cell wall building blocks	Inhibit protein synthesis by binding to ribosomes					
Inhibition	Bactericidal	Bactericidal	Bacteriostatic	Bacteriostatic	Bacteriostatic	Bacteriostatic	Bacteriostatic
Examples	Vancomycin, teicoplanin, eremomycin	Streptomycin, gentamicin C1a	Chlortetracycline, tetracycline, doxycycline, demeclocycline	Erythromycin, clarithromycin, azithromycin, telithromycin	Chloramphenicol	Lincomycin, clindamycin	Pritinamycin, quinupristin, dalfopristin

**Group**	**Oxazolidinones**	**Quinolones**	**Fluoroquinolones**	**Aminoacridines**	**Rifamycins**	**Nitroimidazoles**	**Cephalosporins**

Sub-structure	N-heterocycle, lactone, fluoride	N-heterocyclic quinone	Piperazine fluoride quinone		Napthalenic lactone in macrocycle	Pentene, N-heterocycles	β-lactam with adjoining thiohexacyclene
MOA	Bind to 50S subunit	Inhibit topoisomerase enzymes		Intercalate with DNA, toxic to humans	Inhibit RNA polymerase	Inhibit protozoa and anaerobes	Cell wall, transpeptidase inhibition Bactericidal
Inhibition	Bacteriostatic	Bactericidal	Bactericidal	Bactericidal	Bacteriostatic	Bactericidal	
Examples	Linezolid	Nalidixic acid	Cirofloxacin, enoxacin, ofloxacin, levofloxacin, moxifloxacin	Proflavine	Rifamycin B, rifampicin	Metronidazole, nitrofurantoin	Cephalothin, cefalexin, cefazolin, cefoxitin, cefuroxime, cefotaxime, ceftazidime, ceftrioxone, cefpirome

**Group**	**Ionophores**	**Cyclic lipopeptides**	**Sulfonamide**	**Sulfones**			

Sub-structure	Macrocycle with hydrophobic semi-circle		SO2NH1	SO2			
MOA	Act on plasma membrane, disrupt ion channels		Inhibition of dihydropteroate synthetase				
Inhibition	Bactericidal	Bactericidal	Bacteriostatic	Bacteriostatic			
Examples	Valinomycin, gramicidin, polymyxin B	Valinomycin, daptomycin	Sulfamethoxazole	-			
							

In most antimicrobial research protocols, such conventional antibiotics are included in assays as a positive control, not merely to convey a contrast of efficacy to the study but also as an internal validation of correct execution of the protocol. More importantly, since research on alternatives now embraces the possibility of adjuvancy to counteract resistance mechanisms against these frontline antibiotics, it is important to have a clear understanding of their mechanisms to guide selection of antibiotics for synergism-antagonism testing.

**FIGURE 1:**
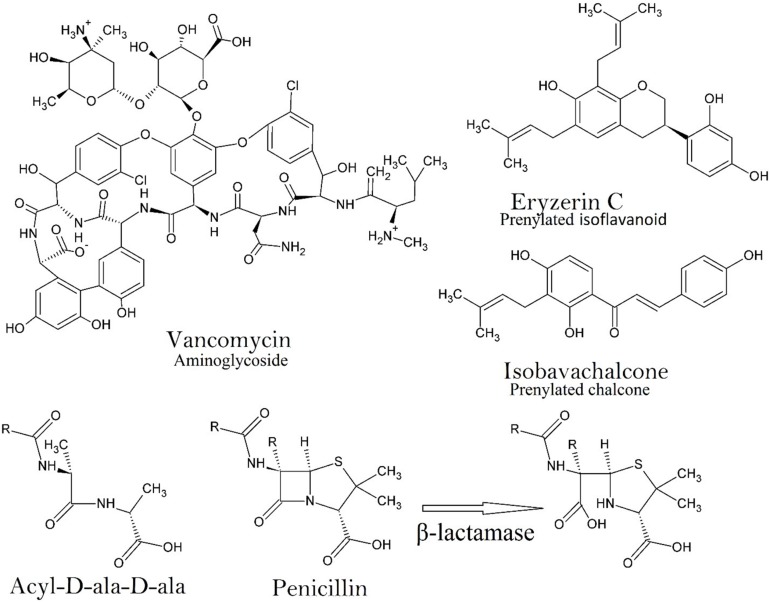
An example of the chemical structure of an aminoglycoside (Vancomycin) showing the complexity of just one of some of the frontline antibiotics. Two structures are depicted that are representative of category 1 antimicrobials (Eryzerin C – prenylated flavonoid; Isobavachalcone – prenylated chalcone), which are among the most potent plant derived nitrogen deficient antimicrobial compounds in nature. The prenyl group enhances lipophilicity and bacterial membrane penetration, the adjacent phenolic OH group (on the same ring as the prenyl group) is essential for efficacy. The structural similarity of acyl-D-ala-D-ala to penicillin is important for the specificity of penicillin since human proteins have no D-amino acids. The activity of β-lactamase against penicillin is on the β-lactam moiety, which hydrolyzes the amide bond (Patrick, 2013).

An appreciation of structure-activity relationships draws attention to the prevalence of amine functional groups and amine-alkaloids (Figure 1) that emerged from the ‘golden era’ as antibiotics with a high degree of specificity. This is not a coincidence. Not only do amine groups enhance solubility whilst retaining lipophilic character (by easy equilibration of ionized and non-ionized forms) but they are often involved in the drug’s binding interactions with its target through specific hydrogen bonding and/or formation of salt bridges.

The high degree of specificity of penicillin comes from its ability to mimic the dimer of D-alanine (D-Ala-D-Ala), a dipeptide amine used in bacterial cell wall synthesis (Figure 1). Other classes of antibiotics include compounds that can disrupt protein synthesis by binding to the 30S or 50S ribosomal subunit, preventing either the reading of mRNA, or translocation or binding of aminoacyl-tRNA (streptomycin, tetracycline, macrolides). At pH 7.4 (homeostatic pH), the cationic amine groups of many classes of antibiotic give them binding affinity to negatively charged pockets in RNA, rRNA or auto catalytic ribozymes (Jia et al., 2013). Antimetabolite drugs such as the sulfonamides, which mimic *p*-aminobenzoic acid, bind irreversibly to dihydropteroate synthetase and prevent biosynthesis of tetrahydrofolate. Ionic interactions of amine groups with various negatively charged pockets in the bacterial membrane also occur, creating pores that enable hydrophilic aminoglycosides to enter the bacterial cytoplasm. Thus, the importance of the amine groups in specificity is evident.

Natural product screening for antimicrobial compounds may conveniently be broken into two major categories; (1) Nitrogen-deficient compounds constructed of C, H, and O atoms (oxygenated hydrocarbons) or C and H only (hydrocarbons), where generalized activity is expected. Specific modes of action are less common but have been reported for aromatics, such as chalcones or flavonoids (Figure 1); and (2) Nitrogenous compounds constructed of C, H and N (and O) atoms (alkaloids, amines, amides, anilines and imines) or C, H, S, O and N atoms (sulfonamides), where the possibility for absolute specificity exists. It is common for compounds in the second category to be synthesized from natural product scaffolds in the first category.

#### Nitrogen Deficient Compounds

In this first category, constituting the predominant class of compounds isolated from plant species, in most (but not all) cases generalized activity against bacterial cell walls or membranes is the expected outcome. It is obvious that simple terpenes or phenylpropanoids, such as those in essential oils, typically demonstrate only low to modest antimicrobial activity attributable to perturbations of the lipid fraction of the cell membrane, enhancing permeability and spilling cellular contents or enabling entry into the cytoplasm (Trombetta et al., 2005). While such activity is, at first blush, unimpressive, such therapies are finding place as topical adjuvants or alternatives to antibiotics. As alternatives, they may mitigate the selective pressure on antibiotics and buy time before resistance development. As adjuvants, sometimes additive or synergistic effects occur, but also on occasion these small lipophilic compounds may antagonize resistance mechanisms and therefore restore efficacy of antibiotics. This is certainly the case with essential oils and volatiles that inhibit efflux pumps, a mechanism that bacteria have evolved to remove antibiotics from bacterial cytoplasm (Aelenei et al., 2016).

Perhaps the two best performing nitrogen deficient classes of antimicrobial compound with ‘noteworthy’ activity are flavonoids and chalcones (Figure 1). The most potent activity in the literature gives values ranging from 0.06 to 2.4 μg/ml against Gram-positive organisms for prenylated flavonoids and chalcones such as panduratin A and isobavachalcone respectively (Cushnie and Lamb, 2011). In terms of structure activity relationships, prenylated isoflavones and chalcones with aromatic hydroxyl groups adjacent to the prenyl moiety give the most pronounced activity (Cushnie and Lamb, 2011; Mukne et al., 2011). The prenyl group is important since it acts as a lipophilic arm and enhances penetration into the phospholipid membrane while the hydroxyl group accommodates the process by interaction with the polar head group of these lipids (He et al., 2014).

Flavonoids and chalcones are special in that multiple modes of antimicrobial specificity have been claimed, mainly against topoisomerases such as DNA gyrase (topo-II) in *Escherichia coli* (Wu et al., 2013) and topo-IV (Mukne et al., 2011). This activity is similar to the mechanism of action of the quinolones and fluoroquinolones of conventional antimicrobial therapy (Patrick, 2013).

The literature dealing with MOA of flavonoids and chalcones is ambiguous but an examination of structural differences, such as glycosylated and aglycone forms, indicates that a single general MOA is unlikely, due to variations in ability to cross cell membrane interfaces. However, the multitude of proposed mechanisms reported in the literature may be an exaggeration, where factors such as ‘cause and effect’ and issues of aggregation of purified enzymes *in vitro* may complicate the interpretation of data (Cushnie and Lamb, 2011). Whilst the possibility of multiple MOAs across flavonoids or chalcones in general is realistic, more comprehensive testing is necessary to confirm this. This should include screening a group of flavonoids or chalcones across a diverse range of MOA assays.

In this context it should be noted that evolutionary pressures would likely select for biosynthesis of secondary metabolites that confer antimicrobial activity via mechanisms unfavorable to resistance development. Drugs with multiple MOAs yield a similar outcome to combination therapies, in that single mutations in microbes are unlikely to create comprehensive resistance mechanisms against multiple targets.

Many follow-up studies have confirmed some of the MOAs reported for flavonoids and chalcones, such as topoisomerase inhibition (Cushnie and Lamb, 2011). Given the previous discussion on the importance of amine functional groups, it follows that the activity of flavonoids might be enhanced by production of amine derivatives. The validity of this approach was demonstrated in the synthesis of a tricyclic sulfur-amino flavonoid, which demonstrated most impressive inhibition of the Gram-positive species *Staphylococcus aureus* down to concentrations of 0.24 μg/ml (Babii et al., 2016), with the mechanism related to the impairment of cell membrane integrity and cell agglutination.

#### Nitrogenous Compounds

As previously stated, compounds from the second category, containing nitrogen and/or sulfur atoms, have hitherto demonstrated the most pronounced antimicrobial activity, with absolute specificity. These have almost exclusively been isolated from bacteria and fungi, but some studies have reported the isolation of such compounds from plants. Natural quinolone alkaloids were isolated from the fruit of a species in *Rutaceae* and screened for antimicrobial activity and in some cases demonstrated noteworthy activity against Gram-positive species. The structures differed by a homologous series of alkyl side-chain moieties, which significantly impacted on MIC values, with chains within the range of 9–13 carbons as yielding the most pronounced activity, as low as 4 μg/ml (Wang et al., 2013). Such a structure-activity profile suggests that cell membrane penetration is enhanced by the alkyl sidechain.

### On Why Some Antimicrobial Agents Fail

#### Supply Challenges

Most antibiotics in common clinical use are of bacterial or fungal origin. This may give the impression of the intrinsic inferiority of plant-extracted compounds. However, this pattern of prioritization of bacterial and fungal metabolites is more related to logistics than efficacy *per se*. Microorganisms can be cultured and are characterized by rapid growth, which makes the supply aspect of a commercial product non-limiting. By contrast, plant derived metabolites require long waiting periods for maturation of plantations, followed by a complex extraction protocol and generally low yield. This makes supply a limiting factor. Sometimes a valuable alternative to the cultivation of plants is realized, but only if it can be demonstrated that microbial endophytes are responsible for the *de novo* biosynthesis of the relevant plant metabolite. In this case, the endophyte can be isolated and cultured as in classical antibiotic production. Alternatively, genetically modified yeasts may also be used to produce specialized metabolites provided the yeasts themselves are not susceptible to the product or its intermediates.

Although the supply of natural antibiotics is a major logistical concern, *de novo* and semi-synthetic approaches can also be employed to make them commercially viable should the need arise. More fundamental challenges to the efficacy of noteworthy antimicrobial drugs are related to the pharmacotherapeutic obstacles encountered *in vivo*. Thus, pharmacotherapeutic challenges could be related to negative side effects, such as toxicity, or failure to translate *in vitro* activity into useful therapeutic activity because of poor absorption, bacterial resistance or biotransformation in the gut or liver.

#### Pharmacodynamic Challenges

Strictly in the context of pharmacodynamics, resistance mechanisms and toxicity are the biggest problems. While many researchers are now seeking to identify compounds effective against resistant strains, far fewer studies employ synergism-antagonism assays, which may lead to the discovery of antimicrobial compounds that work in combinations. The best example of the success of this approach comes from the synergistic effects of clavulanic acid, a weak antibiotic that is related to penicillin by the presence of a β-lactam ring. Since resistance mechanisms in bacteria now include the induction of the enzyme β-lactamase, which cleaves the β-lactam ring (Figure 1) and inactivates penicillin derived antibiotics, inhibiting this enzyme restores the activity of β-lactam antibiotics. Clavulanic acid is classified as a ‘suicide substrate’ in that the β-lactam site is cleaved by β-lactamase in the usual way, but the presence of an enol ether over the fused heterocyclic ring (O in the place of S) causes the drug to bind to the enzyme irreversibly. Thus, combinations of clavulanic acid and β-lactam antibiotics restores the potency of these drugs. This is currently in clinical practice with a product called Augmentin^®^, which combines amoxycillin and clavulanic acid (Cock, 2018).

Another resistance mechanism is the aminoglycoside riboswitch (Jia et al., 2013), which regulates expression of the anti-aminoglycoside enzymes, aminoglycoside acetyl transferase and glycoside adenyl transferase, in response to binding to the aminoglycosides. The expressed enzymes modify the structures of aminoglycosides and inactivate them (Aghdam et al., 2014). Methods to counteract this resistance mechanism are still under development, but some headway has been made with the realization of unique binding activity of paromomycin, which makes a transient hydrogen bond at 6’-OH with A17, diminishing interactions with more important coding regions of the riboswitch, leading to deactivation (Kulik et al., 2018). Researchers are now looking at paromomycin derivatives as new aminoglycoside drugs (Zárate et al., 2018).

No research has yet been published demonstrating combinations that attenuate the riboswitch resistance mechanism. Research has focused more on efflux inhibition, anti-quorum sensing, anti-virulence and anti-infective mechanisms at sub-MIC concentrations that attenuate both pathogenicity and resistance (Cushnie and Lamb, 2011).

#### Pharmacokinetic Challenges

It is apparent that even the lowest MIC values achieved by natural products is still many folds higher than the possible systemic concentrations achieved *in vivo* for oral therapies (not topical). This implies that the antimicrobial outcomes, no matter how impressive, will not be actualized *in vivo* unless other factors are taken into consideration. One neglected area of research is to examine compound accumulation in specific tissues. Another area of research is to redirect efforts toward immunomodulation either in the context of stimulation or conversely, anti-inflammatory (suppression). This aspect is further explored in the section titled ‘routine absorption and immunomodulatory assays.’

### ‘Potentiators’ to Counteract Resistance

Researchers will refer to drugs that antagonize resistance mechanisms as the ‘potentiator’ (Cock, 2018). Thus, compounds that have poor antimicrobial activity may nevertheless affect the virulence or pathogenicity of microorganisms. For example, antimicrobial assays assess activity against bacteria in planktonic growth (as colonies) rather than as biofilms, which are formed because of quorum sensing activities. Since the biofilm itself, and surface adhesion, confers resistance to the immune response and slows antibiotic activity, antagonism of quorum sensing can reduce virulence. Flavonoids, and polyphenols such as catechins, have demonstrated anti-quorum sensing activities. Furthermore, other virulence and pathogenicity factors are antagonized by many polyphenols and flavonoids, such as sortase inhibition (another enzyme implicated in biofilm formation), urease inhibition (for *Helicobacter* to survive stomach acid), listeriolysin inhibition (for surviving phagosomes and entering the cytosol of host cells) or neutralization of bacterial toxins (reducing pathogenicity) (Cushnie and Lamb, 2011).

Drugs that block efflux pumps are potentiators of antibiotics. The intracellular efflux pumps in bacteria have become increasingly capable of excreting a wide array of antibiotics, with the tetracyclines receiving the most attention. Many examples of efflux inhibitors have been discovered, which often include flavonoids and polyphenols at sub-MIC values (Cheesman et al., 2017).

Compounds that antagonize bacterial resistance, virulence and pathogenicity are evidently good potentiators of both the immune system and antibiotics. Thus, they should be seriously considered as adjuvants to conventional therapies (Cock, 2018). However, compounds that are antagonistic of bacterial resistance development *per se* have received the least attention in antimicrobial research. For example, drugs with multiple modes of action, or combination therapies, antagonize resistance development by maintaining efficacy against mutant strains that develop single resistance mechanisms.

Combination therapies can also combine bactericidal drugs with bacteriostatic drugs to counteract resistance development. Such combinations are also beneficial because immunocompromised patients are fully dependent upon the drug and it is difficult to maintain optimal plasma concentrations of bacteriostatic drugs over the course of the infection. Antimetabolite drugs, such as the sulfonamides, are bacteriostatic (Patrick, 2013), but many flavonoids and chalcones have demonstrated bactericidal activities (Cushnie and Lamb, 2011), so this combination may achieve positive outcomes.

Sometimes combinations achieve synergistically enhanced antimicrobial activity, which means that the MIC value is enhanced by more than the sum of the two activities of each drug combined (greater than the sum of its parts). Alternatively, sometimes there are interactions that antagonize activity. Researchers generally test for these effects in a synergism-antagonism assay (Van Vuuren and Viljoen, 2011). The methodology involves testing the combinations at different ratios across different dilutions to produce a ‘fractional inhibitory concentration index’ (ΣFIC). It is obvious that synergistically enhanced activity in combinations is beneficial, but less obvious that it has the potential for providing a wider gap between MIC values and median lethal dose (LD50), if relevant.

### Toxicity

Several methods for measuring LD50 and LC50 values are in practice for describing a compound’s toxicity. Brine shrimp lethality is for some researchers a first step, giving broad implications for human contact (Sarah et al., 2017) but greater specificity is acquired using mammalian cell lines (Ekwall et al., 1990). It is important that drugs with potent antimicrobial activity have much higher toxicity concentrations as compared to MIC values, since concentrations required to kill bacteria should not be damaging to the host. However, when interpreting toxicity studies, one must be aware of research that specifically tests for toxicity against cancer cell lines without appropriate comparison to non-cancerous cells. Obviously, high toxicity to cancer cells but low toxicity to healthy mammalian cells is a positive outcome in this context.

Unfortunately, without knowledge of, or access to, the biotransformed conjugate of the drug as it would appear in the host after metabolism, it is difficult to comment specifically on the toxicity of a drug, if it is an ingested therapy. In antimicrobial outcomes, some of the activity is maintained in the pre-conjugated form, and sometimes as well after conjugation, but toxicity after conjugation is difficult to control for. The fates of xenobiotics after absorption and transformation provide the most common challenges for understanding the pharmacotherapeutics of the drug and this is the jurisdiction of pharmacokinetics.

## The Core of Pharmacokinetics: Lipophilicity, Hydrophobicity and ‘Bioavailability’

It is no surprise that the vast majority of prospective drug candidates are poorly soluble in aqueous solvents. One pharmaceutical company estimates that 30% of drug candidates have aqueous solubility at <5 μg/ml (Lipinski, 2001). While lipophilicity of a drug is an important factor influencing absorption and distribution into the lipid membranes characterizing many human tissues, at least some aqueous solubility is necessary to enable distribution in and from the human GIT. This issue is illustrated in the intestinal permeability prediction assays, such as the caco-2 cell culture, but this problem is replicated in the human gastrointestinal tract.

While exceptions can be made for compounds of low aqueous solubility that are liquids at body temperature, such as with essential oils, [indeed, melting point was considered a contributor in earlier transdermal models (Magnusson et al., 2004)], solid insoluble compounds are not generally bioavailable. While aqueous solubility and lipophilicity are generally treated as opposites, they are not exactly inversely proportional, especially in fluorinated molecules. There are many examples of compounds that are amphiphilic (high solubility in both), such as saponins, but the lipophilic moiety itself is considered important in bringing about bioavailability.

Drugs that are small and strongly lipophilic are often regarded as having good bioavailability. In contrast, high molecular mass drugs only have good bioavailability if they convey fewer rotatable bonds and an optimal balance of lipophilic and hydrophilic moieties, where lipophilic moieties enable passive *trans*-membrane or *trans*-dermal diffusion and polar groups enact biological interactions. In addition, polar groups enhance aqueous solubility and prevent flocculation in the gastrointestinal tract, which aids absorption.

Despite their considerably higher lipophilicity, their typically small molecular size means that essential oils have good bioavailability, but their high lipophilicity means that they may also cross the blood-brain barrier. This outcome could be favorable if essential oils confer immunomodulatory effects, particularly where anti-inflammatory activities are potentially useful. However, one of the most controversial side-effects of some lipophilic drugs is psychoactivity. Almost all psychoactive drugs have high lipophilicity, some of which occur in essential oils, such as elemicin, a psychoactive phenylpropanoid that also confers anti-inflammatory effects (Sa et al., 2014). Pre-conjugated forms (pre-metabolized forms) of essential oils also rapidly dissolve in the fat tissues, giving a shorter half-life in the first instance and creating a reserve or ‘storage’ of potentially bioactive compounds in the second instance.

Ingestion of aromatic foods over time will lead to an accumulation of essential oils in adipose tissue. For example, grazing on the aromatic fodder plant *Penzia incana* leads to an accumulation of *Artemisia*-type terpenes in the fat tissue of South Africa’s ‘Karoo Lamb,’ which confers a distinctive flavor to the meat when roasted (Hulley et al., 2018) and prolongs the immunomodulatory effects of components such as linalyl acetate (Sa et al., 2013). Another example involves the bioaccumulation of cannabinoids in fat tissues of cannabis smokers. Lipolysis induced by exercise or starvation can provide the user with a recycled ‘high’ (Gunasekaran et al., 2009), a side-effect that occurs together with a range of immunomodulatory effects mediated by agonism of cannabinoid receptors (type 2) (Olah et al., 2017). Strains used for medicinal cannabis have higher yields of cannabidiol and less tetrahydrocannabinol (Rom and Persidsky, 2013) to achieve more positive and less psychoactive effects. Due to their high lipophilicity the cannabinoids have very long half-lives, conferring immunomodulatory effects for sustained periods after smoking.

Thus, the activity of anti-infective drugs with good tissue or fat solubility may be prolonged as they are slowly released back into the host’s circulation from such tissues (Patrick, 2013). The drug is usually released from fat stores at a lower concentration as compared to the active systemic concentration, but repeated drug administration enhances this effect. For example, the anesthetic thiopental is highly lipophilic, so its peak concentration is rapidly decreased due to redistribution to more slowly perfused fatty tissues, at which time it is slowly released from fat storage at subanaesthetic concentrations. However, after repeated doses the fat sink is fuller, and thiopental is then released at an active concentration, which keeps the patient anesthetized. This also occurs with drugs that accumulate within cells, such as the anti-malarial drug chloroquine, which accumulates in white blood and liver cells, reaching concentrations thousands of times higher than in plasma.

As drugs become slightly more hydrophilic their aqueous solubility increases and accumulation in adipose and other body tissues becomes less relevant, but as aqueous solubility continues further issues of absorption become prominent. It is therefore important to be able to judge *a priori* the approximate solubility character of a molecule from the number of polar moieties, before it becomes clear if it has potential as a drug candidate. Other important factors include numbers of rotatable bonds and hydrogen donors/acceptors (closely related to polar surface area). Some rudimentary guidelines will now be given.

## Indications that a Drug has ‘Good Bioavailability’

The classical approach used to judge the bioavailability of a drug was ‘the rule of 5,’ which is a set of 4 guidelines that prescribe numerical parameters as a factor of 5. Hence, 1) molecular weight needs to be <500 Da, 2) there must be less than 5 hydrogen bond donor groups, 3) and no more than 10 hydrogen bond acceptor groups, 4) and a calculated log *P* value of less than + 5 (drug hydrophobicity measurement) (Patrick, 2013).

Today it is clear that a substantial number of bioavailable drugs break this rule of 5; drugs commonly referred to as ‘in the space beyond the rule of 5’ (bRo5 space) (Doak et al., 2014). New parameters for prediction of oral bioavailability now acknowledge that molecular size is insignificant, provided that the polar surface area is ≤140 Å^2^ and that the number of rotatable bonds is fewer than 10 (Veber et al., 2002). Percutaneous penetration is a different matter, where molecular size continues to be regarded as significant, but macromolecules that are rapidly absorbed have also been identified (Pino et al., 2012). Thus, there are ample examples where molecules that are easily absorbed break the modern rules.

Unlike polar surface area, the number of rotatable bonds leading to molecular flexibility, is a parameter that is not so intuitive. However, since molecular flexibility tends to become a limiting factor in larger molecules (≥500 Da), a first assessment for smaller molecules should only be for a compound’s polarity, which is easily judged by the number of polar groups and how polar they are.

**FIGURE 2:**
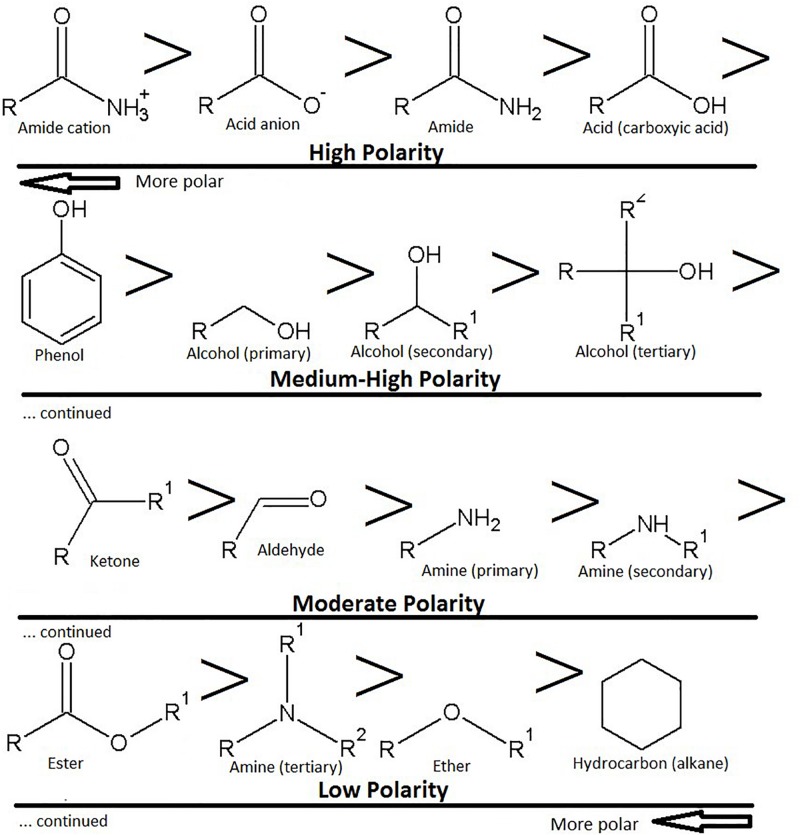
Polarity of functional groups, with the most polar groups as acids and amides, plus their ionic counterparts. The highest polarity groups are toward the top left-hand side of the image, the lowest polarity groups are toward the bottom right hand side of the image. Note that amines can also be ionized.

It is relatively easy to get an approximate estimate of the hydrophobicity of a molecule according to the number of amides, amines, alcohols (hydroxyl), aldehydes, ketones, ethers and acid (ester) groups attached. The order of polarity goes amide > acid > phenol > alcohol > ketone > aldehyde > amine > ester > ether > hydrocarbon (alkane) (Figure 2). For example, sugar groups (glycosides) are pharmacokinetically negative. Generally, when small molecules (≤400 Da) have six or more hydroxyl groups the bioavailability is substantially low. However, monoglycosides (one sugar) are more easily orally absorbed than diglycosides (two sugars), which are better than triglycosides (three sugars) and so on.

By contrast, amines generally have a weaker dipole moment (lower polarity) as compared to hydroxyl groups and have higher bioavailability. They are cationic at pH 7.4 and can interact with the anionic components of the stratum corneum or intestinal epithelium, enabling passage of anionic drugs (Pandey et al., 2014). Surprisingly, small molecules with carboxylic acids and amides are also bioavailable. Accordingly, amino acids are used as penetration enhancers (Sarpotdar et al., 1988), and this effect may be enhanced by esterification of the carboxylic acid moiety (Hrabálek et al., 1994).

**FIGURE 3:**
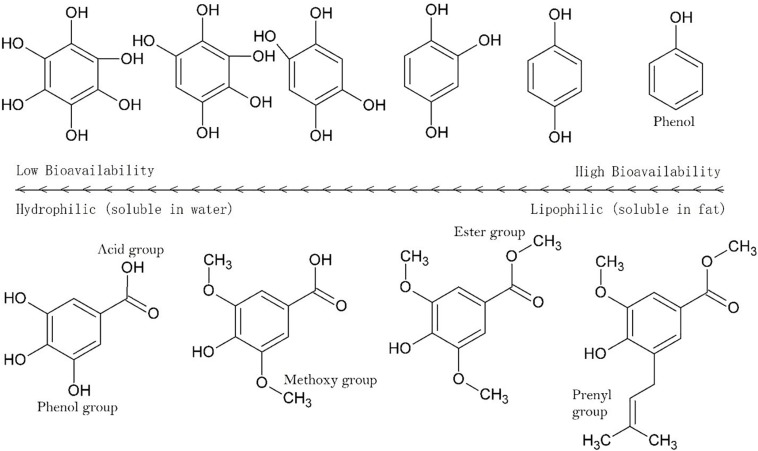
Solubility across a series of hydroxylated aromatics. Hydrophilicity (aqueous solubility) increase with the number of polar groups and the strength of polar groups (see Figure 2). Methylation of OH groups significantly reduces hydrophilicity, as does esterification of acid groups. Similar outcomes occur with acetylation or esterification with alkyl chains, with longer chains having progressively lesser hydrophilicity.

As the numbers of polar groups increase relative to the alkane bonds the bioavailability decreases (Figure 3), decreasing further as the number of rotatable groups increases. The segmentation of molecules into hydrophilic and hydrophobic moieties can enhance penetration across skin, but an even distribution of polar groups on a molecule has the opposite effect. It is not clear if this is the case for intestinal absorption but it is clear that a sugar such as glucose illustrates this implicitly. In sugars there is a 1:1 ratio of carbon to oxygen atoms with each carbon adjacent to an oxygen, five of which are hydroxyl groups. To counteract poor bioavailability, sugars are actively transported in the human digestive tract and are not absorbed across the stratus corneum.

When sugar groups are sterically oriented around a lipophilic triterpene core, this attenuates the interaction of the molecule with the lipid fraction of the epidermis and antagonizes bioavailability. Tannins and saponins are model examples of this effect (Seo et al., 2002; Seeram et al., 2004). Indeed, these two classes of secondary metabolites arguably demonstrate the lowest oral bioavailability in the natural product world and following ingestion are mostly absorbed as much smaller deglycosylated species. Whilst some saponins are absorbed orally it is generally at a very slow rate and rather than crossing into the cell through its plasma membrane, which is comprised by hydrophobic phospholipids, they are either actively transported or enter portal circulation passively by paracellular transport across the tight junction, passing through pores between epithelial cells.

## More on Bioavailability Estimation

While it is convenient to glance at a molecular structure and make tentative predictions about its bioavailability, more comprehensive predictions can be made by following some clear guidelines. As previously mentioned, the number of rotatable bonds (≤10) and polar surface area (≤140 Å^2^) gives the best prediction for oral bioavailability (Veber et al., 2002) and rotatable bonds and molecular weight (≤500 Da) for transdermal or percutaneous ability (skin penetration) (Grice et al., 2010). Although there is much overlap between oral bioavailability and percutaneous penetration, the influence of polar surface area is apparently less significant in the latter. Other differences occur due to active transport mechanisms, which are dependent upon the site of absorption (intestinal space or topical). In the case of compounds that completely break the rules, such as the macromolecules named avicins (Pino et al., 2012), pronounced differences between oral and transdermal bioavailability are likely (see next section).

**FIGURE 4:**
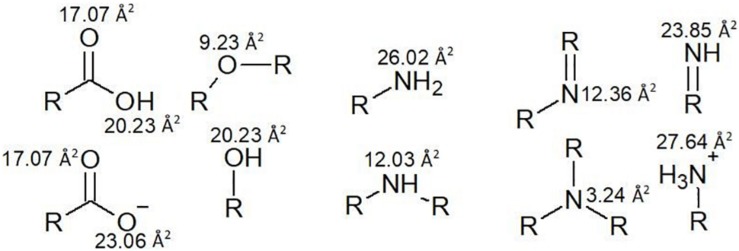
Polar surface areas of the most common polar functional groups.

**FIGURE 5:**
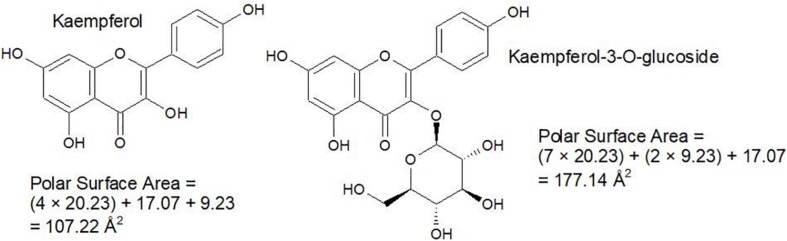
Simple examples for the calculation of polar surface area.

Nevertheless, it is good to be able to accurately predict polar surface area and the number of rotatable bonds. Polar surface area represents the sum of all polar surface areas, including the electronegative atoms nitrogen and oxygen, and their attached hydrogen atoms. These estimations replace the previous convention of the octanol/water partition coefficient (log P). Generally, calculation of polar surface area can take 10 or more minutes per molecule, using specialized software that generates 3D structures *in silico*. Since polar surface area estimations require some time and organization, a cruder strategy which involves counting hydrogen donator and acceptor groups (≤ 12) is used to create *a priori* estimates (Veber et al., 2002). However, use of polar surface area is more accurate, so a faster and more approachable method for this calculation has been proposed by Ertl et al. (2000), which involves assigning standard surface areas to polar groups and summing the values. Some examples from a list of 43 by Ertl et al. (2000) are illustrated in Figure 4 and some examples of the calculation are given in Figure 5.

**FIGURE 6:**
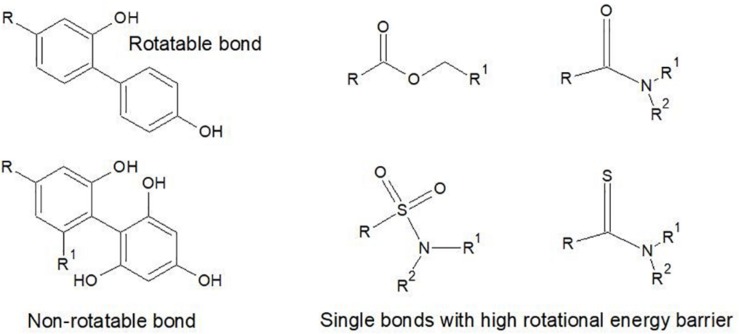
Examples of the types of single exocyclic bonds that are excluded as rotatable bonds due to high rotational energy barrier. In the case of the Ar-Ar bonds, substitution at the ortho positions creates a rotational energy barrier. The S-N and C-N bonds of amides and the C-O bond in esters also have energy barriers against rotation in normal physiological conditions.

For the calculation of rotatable bonds Veber et al. (2002) was a little vague on exclusion criteria, stating only that they are ‘*defined as any single bond, not in a ring, bound to a non-terminal heavy (i.e., non-hydrogen) atom. Excluded from the count are amide C-N bonds because of their high rotational energy barrier*’. Other types of bonds also have a high rotational energy barrier, which means that most chemists also exclude thioamides, sulfonamide bonds, the C-O in ester bonds and single bonds between aromatic groups with three or more ortho substituents. These are illustrated in Figure 6.

## The Pharmacokinetic Journey: From Petri Dish to the Site of Infection

Generally, the pharmacokinetic journey follows the drug up to the site of infection, but then beyond, to the point of elimination. The entire fate of a drug is therefore framed by the acronym ADME, which is an abbreviation of absorption, distribution, metabolism and elimination, as previously mentioned.

The most prominent pharmacokinetic obstacles faced by drugs include transdermal penetration (topical therapies), acid pH of the stomach, digestive enzymes in the human GIT or of bacterial origin, intestinal absorption (oral therapies), first pass metabolism, absorption into the various tissues and organs of the body and blood brain barrier penetration (if relevant).

Thus, oral bioavailability is reflective of the amount of drug in the system after deductions are made for all of these factors, which includes the fraction escaping gut-wall and hepatic elimination (El-Kattan and Varma, 2012). Giving all of these factors some consideration, the school of bioavailability ranks transdermal or gut-wall penetration as the leading obstacle controlling the success of drugs proven *in vitro*, which alone controls the necessary route of administration. Administration routes can be broadly divided into enteral (sublingual, oral, rectal) and parenteral (topical, inhaled, injection). These will now be elaborated upon.

### Parenteral: Topical Therapies

By comparison with ingestive therapies, the efficacy of topically applied remedies for local afflictions is more often reflective of observed *in vitro* outcomes, since compounds are not digested, nor are they subjected to the first pass effect in liver metabolism. Thus, dermal penetration is the only outstanding pharmacokinetic parameter in play, becoming less of an obstacle in damaged or infected tissues.

The pharmacokinetics of topically applied therapeutics is also less complex as compared to ingested drugs. While topical routes are often utilized for administration of systemic drugs expected to act non-locally (e.g., nicotine patch), the following discussion is directed at therapies that target local afflictions, such as pathogenic microbes, infections or inflammation.

Most pathogenic organisms are superficial and easily reached by inhibitory molecules. For example, fungal infections, such as *Trichophyton rubrum*, *T. mentagrophytes* or *T. interdigitalis* (Tinea pathologies), are superficial, since they are external to the dermal layers and can therefore be inhibited *in vivo* as efficiently as observed *in vitro* outcomes. By contrast, the penetration of compounds into an abscess is difficult, even with compounds having high bioavailability. The degree of penetration is influenced by duration of infection and stage of encapsulation (Wagner et al., 2006). Inflammation has also been implicated in changes of drug transport efficiency, with penetration being antagonized in many instances (Schmith and Foss, 2008).

**FIGURE 7:**
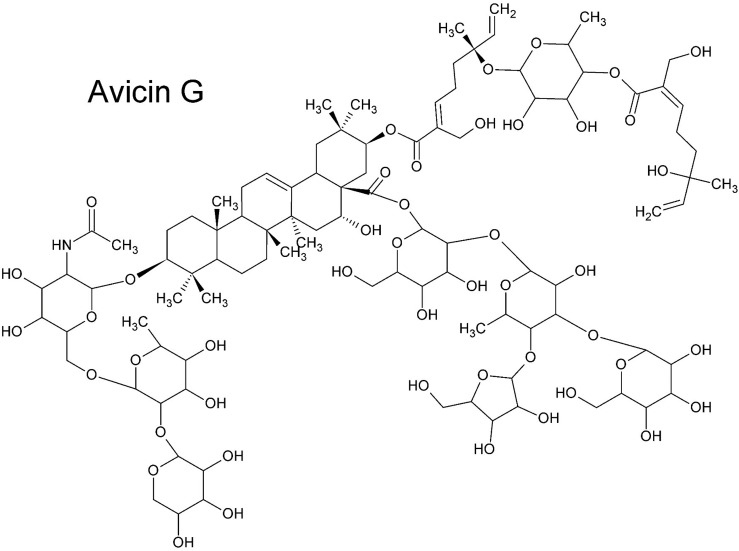
Avicin G (Pino et al., 2012). Despite its large size and the presence of strongly polar functional groups, Avicin G passes epidermal layers as effectively as smaller lipophilic molecules. The steric balance of lipophilic and hydrophilic moieties and the acetamide group is key to the saponin’s transdermal penetrative ability.

Nevertheless, a general rule is that small molecules (≤ 500 Da) that are lipophilic can passively cross dermal layers. As compounds increase in polarity and size the skin permeability substantially reduces. Some larger hydrophilic compounds can also passively cross dermal layers, provided that a low number of rotatable bonds are present. In some cases, polar and apolar moieties are sterically optimized for penetration, but this is not often reported. For example, Pino et al. (2012) discovered that avicins, glycosylated triterpenes (acetamide saponins) with a molecular mass > 2000 Da, pass the dermal layers as rapidly as smaller lipophilic molecules. The key to the structure’s success in this regard is the combination of a geranyl ester moiety (monoterpene), an acetamide group and a tetrasaccharide (Figure 7), conferring both hydrophilic and hydrophobic properties in an optimal spatial (steric) arrangement (Pino et al., 2012).

Poorly bioavailable compounds, such as saponins, may still find a place in topical therapies. Ruptured skin tissue, occurring in conditions such as eczema, provides a privileged passage to important sites. Another privileged passage follows hair follicles, to the dermal papilla, a site of increased permeability with high capillary networking and density of immune cells (Herman and Herman, 2016). Sebum secretions line the inside of the follicle infundibulum, which may be considered a hydrophobic barrier that can be easily breached by lipophilic or amphiphilic compounds, such as saponins. The infundibulum may therefore be a site for the penetration of higher molecular mass compounds with strong detergent-like character.

The fates of compounds in topical therapies as components of complex mixtures applied as extracts are not entirely limited by their individual bioavailability. Other compounds, such as the sterically balanced avicins (Pino et al., 2014) or small lipophilic molecules, can temporarily modify the stratus corneum and allow passive transport of molecules with intrinsically poor bioavailability. Thus, in commenting on the pharmacokinetics of topically applied therapies one must take note of the presence of potential penetration enhancers in extracts. These include high quantities of volatile terpenes (Paduch et al., 2007), phenylpropanoids or others as described by Karande et al. (2005).

### Parenteral: Injection

Due to poor oral bioavailability, or challenges related to modifications by gut microbes, many antibiotics are injected rather than ingested. In the 1950’s it became common knowledge that some antibiotics could be absorbed in the alimentary canal, and some required intramuscular or intravenous injection (Finland, 1958). Those that could be absorbed orally were penicillin, erythromycin, tetracyclines, chloramphenicol and novobiocin. Those that had poor absorption and required injection were streptomycin, neomycin, viomycin, nystatin, vancomycin, ristocetin and various polypeptide antibiotics.

Antibiotics that are absorbed in the intestines can also be given by injection but common practice is to convert to sodium or potassium salts to enhance aqueous solubility. By contrast, those with high aqueous solubility can be injected without conversion. Thus, except for ionized penicillin, the degree of absorption is reflective of aqueous solubility. High aqueous solubility then influences the drug’s ability to reach the target site within the body. This is generally not a problem in antimicrobial research, since most infections are extracellular.

### Enteral: Oral, Stomach Acid as the First Obstacle

At the mastication stage there is the possibility of absorption of lipophilic compounds through the area around and under the tongue, which is known as the sublingual route. In this case absorbed drugs are not subjected to liver metabolism or acid hydrolysis in the stomach. However, the sublingual route is limited, and most patients prefer not to taste their medicines.

Orally administered drugs or therapies generally survive mastication, but it is common practice to preserve drug contents for release further along in the alimentary canal, by encapsulation into a soluble capsule or fashioning into a pill with or without a sugar coating. In most cases pills or capsules release contents into the stomach. Sometimes stomach acids can break down or transform drugs, particularly if electron dense hydrogen acceptors are on the molecule. For example, the first penicillin used clinically was unstable in stomach acid and had to be administered intravenously. Ampicillin is an example of a penicillin derivative that is modified to give resistance to acid hydrolysis in the stomach, by placement of an electron-withdrawing substituent on the α-carbon of the side chain to draw electrons away from the carbonyl group.

Thus, depending upon acid stability, drug capsules can be fashioned for solubility either in the stomach or upon entry into the small intestine. Less commonly employed today, a previously widely used enterosoluble coating came from a gum called ‘sandarac,’ which could be made from the Australian Black Cypress tree (*Callitris endlicheri*) (Sadgrove and Jones, 2014b, 2015), or the actual sandarac tree (*Tetraclinis articulata*).

### Enteral: Oral, GIT Metabolism as the Second Obstacle

In the intestines microbial transformations can also be a significant challenge (the domain of pharmacomicrobiomics). For example, although warfarin survived stomach acid, mixed therapeutic results observed in patients were hypothesized to be related to individual differences in the gut microbiome (Das et al., 2016). Digoxin is another drug that is susceptible to the microbiota, but unlike warfarin, it is consistently transformed into a less active form (Jourova et al., 2016). To curb such effects drugs can be administered together with a general antibiotic to kill off the microbiome and prevent transformations. This is the procedure for the anti-inflammatory drug sulfasalazine (Jourova et al., 2016). Obviously, this latter procedure is undesirable.

By contrast, microbial transformations of non-active ‘prodrugs’ can in some cases create potent antimicrobial drugs. In 1935 it was discovered that the red dye called prontosil had antimicrobial activity *in vivo*, but this was not evident *in vitro*. It was discovered that prontosil was metabolized by bacteria in the small intestine into sulphanilamide by reductive removal of the benzamine moiety. Today, many such prodrugs are known. The most important derivatives come from ampicillin. Lipophilic prodrugs are made by esterification with substitution groups, such as acyloxymethyl or pthalide, to remove the potential ionization of the carboxylate and aid absorption. The ester is subsequently hydrolyzed in phase-1 metabolism to produce active ampicillin.

Nearly all biotransformation reactions occur on the more electronegative atoms, such as oxygen and nitrogen, or with atoms that have non-bonding electron pairs, such as the former two atoms and sulfur, or atoms conjugated to oxygen, such as alpha-beta unsaturated ketones. Thus, in addition to alkenes and α-alkene carbons where hydroxylation commonly occurs, these three heteroatoms are where most of the transformations take place (Patrick, 2013).

**FIGURE 8:**
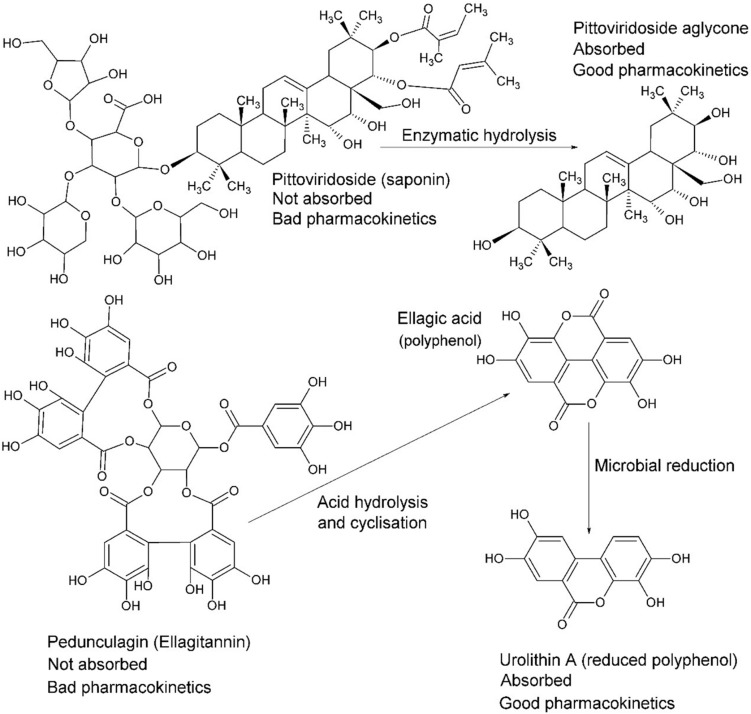
The fate of complex glycosides in human digestion involves cleavage of sugar moieties, either by β-glucosidases of the small intestine (or bacteria) in the case of saponins, such as pittoviridoside (Seo et al., 2002), or by acid hydrolysis in the stomach, such as pedunculagin (Seeram et al., 2004). Ellagic acid is metabolized into urolithin A by gut bacteria before absorption; however, both are systemic. The pentacyclic triterpene of pittoviridoside is absorbed after hydrolysis of the sugar and butenoic ester moieties.

Intestinal passage usually reduces the size of compounds via reductive and/or hydrolytic processes. Whilst reduction tends to increase polarity, the overall outcome may generate higher lipophilicity, such as by dehydroxylation or removal of a sugar moiety (Figure 8; Jourova et al., 2016). For example, in many instances the reductive process of hydrolysis creates aglycone moieties of glycosides (Day et al., 2000), which sees a substantial increase in lipophilic character of the aglycone moiety, making systemic absorption passive (Németh et al., 2003). This process has a most significant potential impact in research on natural products because it is obvious yet neglected. Other significant transformations include hydrolysis of esters and amides, particularly peptides (Patrick, 2013).

Generally, by the time glycosides enter systemic circulation they are present as aglycones or mono-glycosylated glycosides (less commonly di-glycosides). Glycosides are mostly absorbed by active mechanisms in the small intestine (Németh et al., 2003) but larger molecules enter between cells through pores. Lipids are digested to release free fatty acids. Alkyl esters or alkyl amides are often cleaved but can also be absorbed intact, with combinations of both appearing in blood plasma, such as those homologous alkylamides from *Echinacea* (Matthias et al., 2007), which are modulators of the immune system (Raduner et al., 2006).

### Enteral: Absorption as the Third Obstacle

As previously mentioned, polar surface area and the number of rotatable bonds will influence the absorption rate or efficiency of a drug. While most absorption occurs passively, on occasion, metabolites enter circulation via active mechanisms. The major implication is that even strongly polar groups can be absorbed, provided that there is compatibility with one of the transport routes.

Surprisingly, even a small hydrophilic molecule, such as the simple sugar glucose, requires active transport for absorption. It was once thought to be passively absorbed but it has long since been demonstrated that the Na^+^/glucose cotransporter is responsible for absorption in the small intestine (Chen et al., 2016). This has implications for mechanisms of absorption of glycosides in general. Although they are predominantly absorbed passively after deglycosylation (Figure 8), they can be actively transported as monoglycosides on the hexose transport pathway by interaction with the sugar moiety (Gee et al., 2000). Nevertheless, the fate of glycosides that enter the portal circulation is deglycosylation by liver metabolism, but xenobiotics that make the first pass in metabolism may enact biological interactions before phase-1 transformation in the second pass.

A pharmacokinetic study of flavonoid or chalcone aglycones and saponins in rats gives insight into the differences of absorption from the GIT between these two types of compound. Relatively apolar compounds, with fewer hydrogen donor and acceptor groups, reach peak plasma concentration in 5–30 min (Ying-Yuan et al., 2019). A similar outcome was observed with monoglycosides. But the diglycosides demonstrated completely different kinetics, with peak concentrations being seen after 8 h on average. The ginsenosides specifically had long half-lives (12–25 h) but other diglycosides were more similar to the less polar compounds (2–11 h). This study shows that it is definitely possible for diglycosides to enter systemic circulation, albeit much slower by comparison with less polar compounds.

Following absorption, post-metabolized drugs can be returned to the GIT where they are metabolized to their pre-conjugated or phase-1 metabolized state. For example, glucuronidated drugs enter the GIT for excretion but are cleaved by intestinal microbes that express β-glucuronidase enzymes and reabsorbed (Pellock and Redinbo, 2017).

### Enteral: ‘First Pass’ Metabolism

The discipline of pharmacokinetics encourages us to consider the potential biological effects of more polar conjugated drug forms, or ‘xenobiotics,’ as they are metabolized during the body’s elimination response by phase-1 and phase-2 enzymes, such as cytochrome P450 isozymes (phase-1) and/or transferase enzymes (phase-2) such as glucuronosyltransferase. This complex series of enzymatic transformations occurring mainly in the liver may, depending on the chemistry of the original compounds, either enhance or attenuate activity and/or toxicity.

Antimicrobial compounds that are absorbed (now called xenobiotics) and become ‘first pass’ have the capacity to enact biological effects provided they are distributed to the site of infection. In some cases, however, a significant amount of drug will be metabolized before becoming systemic. Phase-1 reactions may continue the reductive and hydrolytic process started in digestion, particularly on actively absorbed glycosides that were absorbed from the intestine before microbial modifications. Thus, much like the microbial processes, nitro, azo and carbonyl groups are the most common sites for reduction, and amides, esters and glycosides are the sites for hydrolysis (Patrick, 2013). The predominant group of enzymes responsible for these phase-1 reactions are the cytochrome P450 isozymes.

**FIGURE 9:**
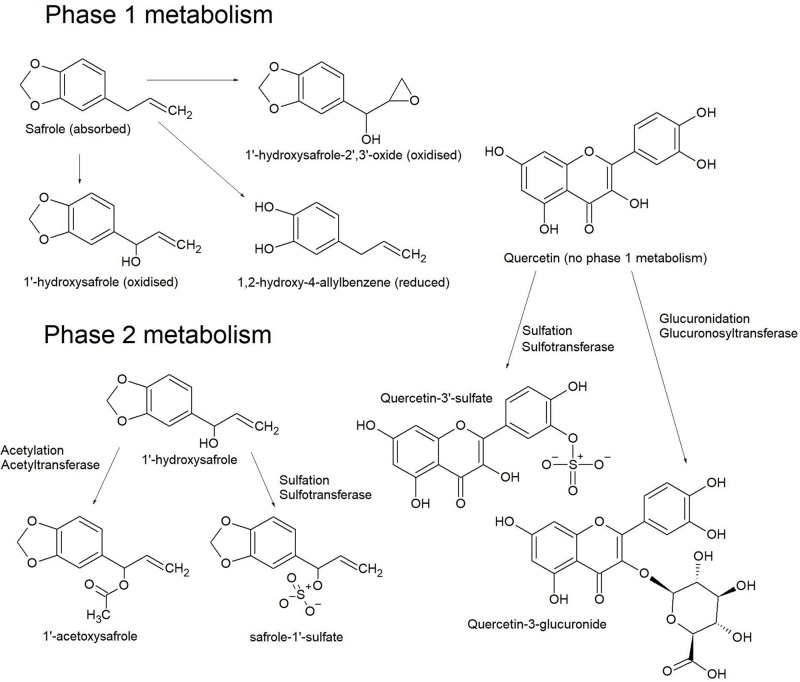
Phase 1 metabolism of safrole produces a number of hydroxylated derivatives, but quercetin is less commonly derivatized at this step. Phase 2 metabolism of 1’-hydroxysafrole produces acetoxy and sulfate derivatives where the hydroxyl ‘handle’ from phase 1 metabolism is used to attach the respective conjugates. The two most common conjugates on quercetin are a sulfate at the 3’ OH or at the 3 or 7 OH for glucuronic acid (O’Leary et al., 2003).

In the liver, phase-1 reductions are less common than oxidative processes. Oxidations typically occur on N-methyl and aromatic groups, thiols, the terminal position of alkyl chains and sterically favorable positions on an alicyclic moiety. These reactions commonly put OH groups on carbon atoms (hydroxylation) (Figure 9) and aliphatic primary amines; oxygen anions on N-methyl groups or N-heteroaromatic rings; keto groups on thiols, with conversion of thiol amines to sulfonamides and so on. Such reactions are said to create a ‘handle’ for subsequent phase-2 oxidative processes (Patrick, 2013).

Phase-2 oxidative processes use the ‘handle’ created in phase-1 reactions (Figure 9) to attach a strongly polar group. These are mostly conjugation reactions catalyzed by transferase enzymes, installing a polar group such as sulfate, O-glucuronide, C-glucuronide, glutathione and less commonly apolar groups, such as methyl groups or cholesterol. Conjugated xenobiotics are usually inactive but sometimes toxic compounds are created (Patrick, 2013).

By far the most common transferase reactions are glucuronidation and sulfation (Figure 9), particularly with aromatics such as flavonoids (O’Leary et al., 2003). In this reaction glucuronic acid is conjugated to phenols, alcohols, hydroxylamines, carboxylic acids, amides, amines and thiols. Sulfate conjugation also occurs on phenols, alcohols and amines. Furthermore, carboxylic acids (and cleaved esters) can be conjugated to cholesterol or amino acids. Glutathione conjugates form with the nucleophilic thiol group of the tripeptide moiety by reactions with electrophilic functional groups, such as epoxides, alkyl halides, sulfonamides, disulfides and radical species. Glutathione conjugates can also be transformed to mercapturic acids.

Some conjugation processes decrease the polarity of xenobiotics, such as methylation or cholesterol esterification. While methylation often happens in conjunction with glucuronidation (Williamson et al., 2005), it is also possible that some of the transformations are not specifically designed to remove metabolites. For example, at pH 7.4 some flavonoids are stabilized by glucuronidation, which then can be actively transported across cell interfaces by de-glucuronidation and re-glucuronidation at convenient locations. Less frequently, some conjugated xenobiotics such as flavonoids and chalcones (Williamson et al., 2005), maintain bioactivity consistent with pre-conjugated forms. For a drug to be approved for use in therapies it is a requirement that all the potential conjugated forms, including enantiomers, epimers and other isomers, be screened for biological activity, testing for efficacy or toxicity. However, all possible conjugates will be an overestimate. In due course the actual conjugate forms will be revealed by *in vivo* studies that screen blood plasma and urine for the xenobiotics on their exit from the host. Such studies are the only real way to know of the relevant conjugate forms for any particular drug.

Nutraceuticals, functional foods and complementary therapies (including Traditional Chinese Medicine, TCM) do not require this level of approval and *in vivo* studies can be conducted as a preliminary step to identify the conjugated forms, which can be synthesized or isolated in follow-up to screen for bioactivities and toxicity. In such pharmacokinetic studies the pre-conjugated form of the absorbed compound is detected, together with biotransformed derivatives representative of both phase-1 and phase-2 metabolism.

### Circulation to the Site of Infection

Once a drug enters systemic circulation it will easily go into the fluid surrounding the various tissues, cells and organs, since extracellular space is relatively porous, allowing passage of most molecules that are smaller than proteins. In some cases, drugs bind to plasma proteins and are slow to leave the systemic circulation. In a similar way to the adipose ‘sink,’ protein bound drugs are slowly released as plasma concentrations reduce.

In general, a drug’s lipophilicity influences its ability to penetrate a mammalian or bacterial cell wall. For example, streptomycin is evidently strongly polar, a consequence of the trisaccharide moiety. To overcome the issue of absorption, and deglycosylation in the intestines, streptomycin is therefore given by parenteral routes, but since its mode of action is against the bacterial ribosome, absorption across bacterial cell wall and membrane interfaces must occur. Indeed, this is the case, with a cationic charge in alkaline environments the aminoglycoside interacts with the outer membrane of Gram-negative bacteria and displaces magnesium and calcium ions, disrupting ionic bridges and creating pores through which the drug enters the cytoplasm.

## Lessons for Ethnopharmacology

The presumed route of therapeutic application influences how closely the outcomes of *in vitro* anti-infective studies will be reflected *in vivo*. For example, inhaled therapies for bronchial afflictions are expected to closely reflect *in vitro* outcomes, provided only the volatiles are screened, including hydrodistilled essential oils and/or other volatiles with moderate aqueous solubility that dissolve into the hydrosol. In the case of ‘smoked’ therapies a wider selection of volatiles than those present in essential oils or hydrosols are expected to be relevant. For example, often diterpenes, amines, pyranocoumarins, cannabinoids or drimane sesquiterpenes (Khumalo et al., 2018) are present in acrid steamy smoke. These larger compounds are not present in essential oils because they require higher temperatures than those employed in hydrodistillation to become volatile (Sadgrove et al., 2016). Furthermore, heat derived artifacts that dramatically increase the antimicrobial activity, such as genifuranal (Sadgrove et al., 2014a), are a rare occurrence, but if observed should nevertheless be included in antimicrobial assays.

In ethnopharmacology there is a pronounced difference between topical and ingestive therapies, particularly in the context of anti-infectives. Since it is so common for antimicrobial therapies to demonstrate *in vitro* activity that is only moderate, it is only practical to interpret contact inhibition in the context of topical applications. Where ingestive therapies are being studied, a failure to identify highly potent antimicrobial active compounds encourages us to examine immunomodulatory activities in interpreting their presumed therapeutic value.

However, ingestive therapies are inherently more complex to interpret. Often the fate of natural products is to be judged as toxic, due to inhibition of cytochrome P450 isozymes. Unfortunately many natural products, nutraceuticals and herbs are considered as either toxic or as having the potential for negative interaction with pharmaceuticals (Sasaki et al., 2017). Nevertheless, these studies often neglect the poor absorption of ‘toxic’ components and fail to screen metabolized forms. Alternatively, natural therapies that contain compounds that confer cytochrome P450 inhibition may also be considered as adjuvants in the context of enhancement of drug half-life. Such adjuvancy is seen to be important in the administration of antiretroviral therapies (Dresser et al., 2000). Thus, such interactions must be considered more broadly both for their potential adverse and/or beneficial effects.

### Routine Antimicrobial Assays

Two methods are commonly employed to generate *in vitro* antimicrobial outcomes, the first being the disc (or disk) diffusion assay and the second more precise method, the two-fold serial broth dilution assay, which generates a minimum inhibitory concentration (MIC) value in a 96-well microtiter plate.

The classical ‘disk diffusion’ is a well-established method for screening of antibacterial activity, using an absorbent paper disk that is loaded with an extract or purified compound and placed on the surface of a petri dish containing a medium (usually agar based) inoculated with a test organism. After incubation for 24–48 h, the diameter of the clearing zone around the disk reflects the inhibitory power of the sample. The simple disk diffusion protocol is no longer regarded as the preferred method but continues to be used today as a pre-screening tool to aid prioritizing samples for the more conventional method of testing.

Thus, today the standard *in vitro* method for measuring antimicrobial activity is the two-fold serial broth dilution, minimum inhibitory concentration (MIC) assay (Eloff, 1998; Andrews, 2000) that is now controlled by the Clinical Laboratory and Standards Institute (CLSI, 2017). While this approach measures bacterial inhibition of an extract or compound, the protocol can be made more comprehensive by determining bactericidal concentration (MBC) in subsequent steps. MBC receives the least attention in antimicrobial research, since most antimicrobial natural products are bactericidal, due to generalized MOA. It is less common to find drugs that are bacteriostatic only (like tetracycline), thus, MIC and MBC concentrations are usually within proximal range.

The MIC assay is a serial dilution method, where the concentration of pure compound or complex mixture screened against microbes is successively half the previous concentration. For example, starting at 32 μg/ml, tetracycline dilutions will be 32, 16, 8, 4, 2, 1, 0.5, 0.25 μg/ml et cetera. Problematically, it is common for reviewers to demand standard deviations. The data is ordinal not continuous and so standard error or deviation is not meaningful, but it is acceptable to represent the data as an average of individual assays. Furthermore, MIC values represent an upper maximum, with the actual value in between the MIC value and the next dilution.

Since data is reported as μg/ml, the MIC value is not representative of the actual efficiency of the compound, as it would be if represented as molarity or molecules per CFU. For example, since bacterial cell density is diluted to approximately 5 × 10^5^ CFU/ml, a compound with MIC value of 1 μg/ml and mass of 444.5 g/mol inhibits with 2.7 × 10^9^ molecules per CFU, but for a compound with double the mass, with the same MIC value, it is 1.4 × 10^9^ molecules per CFU, apparently showing double the efficiency.

It is a common fallacy that the inclusion of frontline antibiotics as a positive control is to show the efficacy of a drug in the context of the expected outcomes from the pharmaceutical industry, with positive control and treatment drug as competitors for the lowest MIC value. Whilst such a comparison is important, expected MIC values from leading antibiotics are widely known (Andrews, 2000). The inclusion of a positive control is more about validation of the experimental protocol as executed by the researcher by comparing results against expected outcomes. This is to provide a higher level of standardization and quality control to create more realistic and confident comparisons between research outcomes from different laboratory environments. Whilst low MIC values are of value, it is more important to consider the toxicity of a new drug, its pharmacokinetics and the logistics of administration. This is because the true efficacy of an antimicrobial therapy lies in the quantities that can be safely administered to achieve optimal MIC plasma concentration and its ability to be circulated to the site of infection. If it is a safe and cost-effective drug, that is easily absorbed, such as a flavonoid, then it is theoretically better than a drug with an exponentially lower MIC value that has a narrower toxicity threshold.

For example, purely in the ethnopharmacological context, it is a common mistake to judge only the activity of the molecule *in vitro* and not consider its relative abundance in the extract or raw plant material. However, from a traditional practitioner’s perspective, high concentration of a compound with medium activity is better than very low concentration of a compound with noteworthy activity. An example that illustrates this concerns the volatiles from *Eremophila longifolia* (Sadgrove et al., 2011), a species that is comprised of many individual chemotypes. Some chemotypes have extremely high yields of essential oils (isomenthone and menthone) that have only low antimicrobial activity, but other chemotypes have low yields of essential oils (containing borneol and α-terpineol) that have more moderate activity. Topical use of either chemotype will likely produce a similar antimicrobial outcome. Systemic circulation is not important in this context since the extracts are applied directly to the site of infection and transdermal penetration is all that is required. In ethnopharmacological studies, provided that active antimicrobial ingredients (or active combinations) are extracted by the traditional method at a higher concentration than the value given by MIC, then bacterial or fungal inhibition is possible.

However, where systemic concentrations are relevant, such as where oral therapies are used, it is often the case that *in vitro* values are not possible, yet anecdotal accounts continue to argue in favor of the efficacy of the botanical therapy. In such cases, it is conceivable that other mechanisms can explain infectious control. In this regard, there has not yet been enough interest invested in immunomodulatory compounds.

### Routine Absorption and Immunomodulatory Assays

A vast number of assays are used to create *in vitro* estimates of bioavailability and immunomodulation, but researchers often use animal skins (pig or rat) for transdermal measurements and human epithelial colorectal adenocarcinoma cells (Caco-2) or jejunum *ex vivo* for intestinal absorptivity measurements (Angelis and Turco, 2011).

Outcomes from animal skin models are reported as either maximum flux (*J*_max_ in mol/cm^2^ per hour) or a permeability coefficient (*K*_*p*_ in cm.s^–1^). The permeability coefficient merely gives rate but of more importance is the maximum flux (*J*_max_) because it denotes the quantity of drug absorbed (Grice et al., 2010). The maximum absorbable dose (MAD) for Caco-2 and jejunum permeability is also given as a *J*_max_ value.

Challenges to absorptivity occur in cases of poor aqueous solubility, which can pose a significant problem to the intravenous and ingestive approach to drug delivery; but this does not negate the possibility of transdermal absorption, since solubilizing agents and penetration enhancers can be used in topical applications. Many transdermal penetration enhancers are known and include a long list of essential oil ingredients (Aqil et al., 2007; Chen et al., 2015), triterpene glycosides (saponins) (Shastri et al., 2008), other surfactants (Pandey et al., 2014), amino acids (Sarpotdar et al., 1988) and esters of omega amino acids (Hrabálek et al., 1994), just to name a few.

As previously mentioned, inflammation can antagonize bioavailability of topical antimicrobial drugs, with the encapsulated boil (abscess) being the clearest example. Thus, anti-inflammatory activities not only favor symptomatic relief but expedite the activity of anti-infective drugs by enhancing tissue penetration.

Animal (*in vivo*) and *in vitro* methods are commonly used to predict anti-inflammatory activity, either by direct observation of inflammation or measurement of inflammatory markers, regulatory proteins or pro-inflammatory cytokines. This follows the deduction that inflammation can be prevented either by inhibition of regulatory proteins or binding to pro-inflammatory cytokines.

The most common inflammatory pathways considered *in vitro* include mitogen activated protein kinase (MAPK) and nuclear factor kappa B inhibitor alpha (IkB-α), which are activated when phosphorylated (Harbeoui et al., 2019), leading to release of MAPKs or nuclear factor kappa B (NF-kB) respectively, signaling expression of pro-inflammatory cytokines such as tumor necrosis factor-α (TNF-α) and interleukins (IL-β, IL-6). Another inflammatory pathway is controlled by cyclooxygenase (COX) isoenzymes, which mediate expression of the lipid-based prostaglandins, such as prostaglandin E_2_ (PGE_2_) (Ricciotti and FitzGerald, 2011; Nile and Park, 2013). This latter pathway is better known in the context of arthritic pain.

Markers of inflammation also include nitric oxide (NO), inducible isoform of nitric oxide synthase (iNOS), 15-lipoxygenase (15-LOX) and hypoxanthine oxidase or xanthine oxidase (HX/XO) (Nowakowska, 2007; Harbeoui et al., 2019; Lawal et al., 2019) leading to secretion of the superoxide anion in the latter. These are more often measured to predict the level of inflammation, rather than explain cause and effect.

The most common *in vitro* anti-inflammatory biomarkers reported in the literature describe downregulation of the proinflammatory cytokines (TNF-α, IL-β, IL-6), markers of inflammation (NO, iNOS, 15-LOX, HX, XO), hormones (PGE_2_) or regulatory proteins (MAPKs, NF-kB, COX-2).

A common *in vitro* method to make such prediction uses lipopolysaccharide (LPS) induced macrophages, such as the RAW 264.7 cell line (Harbeoui et al., 2019) and measuring attenuation of any of the above inflammatory markers. In the natural product world, studies often show that species with proanthocyanidins (Lawal et al., 2019) and chalcones (Nowakowska, 2007) often demonstrate attenuation and may therefore have anti-inflammatory effects, provided that the chemical species are bioavailable in the first instance and in the second, demonstrate the same activity *in vivo*. Thus, it is important for researchers to not only be aware of *in vitro* outcomes from studies of poorly bioavailable compounds, but to take into consideration the possibility of multiple activities of the compound. For example, often phenols (flavonoids, chalcones) non-selectively bind to all or most free enzymes. Without selectivity (or absolute specificity) the perception of anti-inflammatory activity is unlikely to be vindicated *in vivo* because of problems of acute toxicity, which compromise potential therapeutic effects.

Even at the very low concentrations demonstrated *in vitro* for antimicrobial or anti-inflammatory therapies, it is often the case that the same concentrations are not reached *in vivo* when administered orally. In such cases presumed efficacy may possibly be explained by such phenomena as bioaccumulation and concentration in source tissue (lipophilic actively transported) or another mechanism altogether involving immunomodulation as previously mentioned.

One exciting area of research that has not yet received enough attention is cannabinoid receptor-2 agonism (CB_2_) (Rom and Persidsky, 2013). While CB_1_ is associated with the psychoactive effects of cannabis smoking, the CB_2_ receptor mediates anti-inflammatory and immunomodulatory activity. However, activation of CB_2_ is considered immunosuppressive rather than the converse (Olah et al., 2017). Almost counterintuitively, immune-stimulating compounds are generally proinflammatory (Kang and Min, 2012; Tsai et al., 2018) but can do so at substantially lower systemic concentrations than required for direct antimicrobial effects. Other neglected areas of research include insulin mediated T cell stimulation (Tsai et al., 2018) and toll-like receptor agonism (Chen and Yu, 2016). The latter, toll-like receptors, have 13 known types to date and are present on various immune cells as innate pathogen recognition defense mechanisms. Fortunately some information on toll-like receptor agonism by natural products can be garnished from the literature (Chen and Yu, 2016), but it is clear that a paradigm shift in the natural products world is called for, where natural products are screened for toll-like receptor agonists particularly type 4, in conjunction with the continuing effort to find antimicrobial candidates.

### Ingestive Therapies

Some of the important pharmacokinetic factors introduced in this review that are most neglected in ethnopharmacological studies include the effects of biotransformation of ingested therapies, synergisms and antagonisms of mixtures and the possibility of chemical variability (chemotypes) of the botanical species studied.

The occurrence of chemotypes in medicinal species, if not recognized, can compromise the reproducibility of bioassays. Plants often demonstrate high degrees of intraspecific variability of secondary metabolites. For example, the Australian species *Eremophila longifolia*, highly regarded as an anti-infective medicinal plant by indigenous populations, is known to have at least 10 different chemotypes (Sadgrove and Jones, 2014a). Furthermore, species may have taxonomic issues, with heterogeneous species aggregates complicating taxonomic determination, which will inevitably introduce chemical variability (Sadgrove et al., 2014b). Thus, the results of studies that screen crude extracts without further chemical characterization of the components can fail the test of reproducibility in subsequent research. Alternatively, if an active ingredient is identified the effects of chemovariability can be elucidated and issues with reproducibility can be explained.

Where chemical studies are undertaken, as previously mentioned researchers often measure antimicrobial activities against compounds that are evidently not absorbed or are entirely broken down in digestion. For example, many South African medicinal barks, or tubers, that are high in hydrolyzable and condensed tannins are used to target gastrointestinal pathologies (Van Wyk et al., 2009). Tannins are non-specific protein poisons and, at the concentrations extracted by these medicinal plants, will not only erase the gut microbiome but will also knock out an epidermal layer. However, only condensed tannins will get as far as the small intestine since hydrolyzable tannins are destroyed in the acid pH of the stomach and broken into their component phenolic acids and sugars (Figure 8). Condensed tannins and some phenolic acids are poorly absorbed but since the site of infection is in the gut, gastrointestinal pathologies are easily antagonized by these therapies.

In a similar way to tannins, ingestion of saponins above a certain threshold can also kill off the gut microbiome before passing out in the stool, mostly undigested. But ingestion to achieve saponin concentration below inhibitory concentration accommodates digestion into aglycones (Figure 8), which are absorbed more efficiently than the saponin itself. Thus, studies that elucidate biological roles for saponins must be considered in the context of topical versus internal application as well as dosage.

For example, dried and pulverized leaves of the Australian medicinal plant *Pittosporum angustifolium* are widely traded on the ‘underground complementary therapies market’ with claims of anticancer activity following ingestion (Sadgrove and Jones, 2014c) together with effective reversal of gastrointestinal pathologies. Inspired by such claims, studies examined cytotoxicity of the main saponins against cancer cells, demonstrating a positive outcome (Bäcker et al., 2014a, b). These saponins are very large, with four or more sugar units, meaning they are barely absorbed. It is likely that the O-linked sugars are hydrolyzed and only the aglycone is absorbed or its monosaccharide form. Thus, it makes more sense to screen the triterpene aglycone moieties for bioactivity, since triterpenes generally demonstrate positive outcomes across a range of pathologies (Yamai et al., 2009). However, at one stage herbalists were prescribing impractically high quantities, which would kill the microbiome, thereby preventing digestion of the saponins. Nevertheless, if they were targeting a gastrointestinal pathology it is useful in the short-term to ingest such high quantities. However, chronic consumption at such high concentrations may be contraindicated, since the microbiome has importance in digestion and indeed in modulating the immune response.

Anti-infective compounds that are not absorbed could be given via parenteral routes to target non-local infections. But high systemic concentrations of saponins is likely to be dangerous, due to the hemolytic (detergent-like) effects. Such hemolytic effects may explain the moderate antimicrobial activity of aqueous leaf extracts of *P. angustifolium* against Gram-positive organisms, which has little relevance for pathologies requiring absorption (Sadgrove and Jones, 2013). By contrast, the traditional use of *P. angustifolium* is most commonly topical, for amelioration of eczema where infective microorganisms could be a comorbidity (Sadgrove and Jones, 2013). Thus, *in vitro* bioassays of the saponins could be informative for topical applications, whereas extrapolation to use as an ingested therapy is problematic. It is therefore better to look at possible immunomodulatory effects occurring at lower concentrations, mediated either by the saponin itself or the aglycone moiety.

A pharmacokinetic study of ingestion of Chinese herbs demonstrated that the monoterpene and flavonoid glycosides were completely absent in the serum of human candidates and the respective free aglycones were only present in trace amounts, with dominant conjugated forms as sulfates and glucuronides (Figure 9; Lee Chao et al., 2006). It is common for some flavonoids to be absorbed as glucosides, the most prominent being quercetin-3-glucoside (Németh et al., 2003). The mechanism of absorption is active, via the glucose carrier SGLT-1 across the brush border membrane of the small intestine (Wolffram et al., 2002).

A similar study of the stevia glycosides demonstrated that only the aglycone diterpene ‘steviol’ was absorbed in both rats and humans and was dependent upon the cleavage process in digestion (Koyama et al., 2003), which was then metabolized to steviol glucuronide. The steviosides are evidently hydrolyzed by the gut microbiome, a process that requires β-glucosidase enzymes. Although the steviosides are more popularly known as natural alternatives to sugar for sweetening of beverages, these diterpene glycosides have been recognized as conferring anti-inflammatory and other immunomodulatory effects (Boonkaewwan and Burodom, 2013), wherein the activity of the metabolite steviol was more pronounced than the glycoside.

Most natural glycosides contain β-glycosidic linkages, which are easily cleaved by enzymes secreted by gastrointestinal bacteria. However, it is also common for the host plant to have β-glucosidase isozymes (Boonclarm et al., 2006). There is good evidence that moderate heating of a mixture of herbs within the range of 40–70°C can influence flavonoid β-glycosides and β-glucosidase, driving enzyme activity, which produces aglycones, but the enzyme denatures at higher temperatures (Zhang et al., 2014). In ethnopharmacological research, therefore, care must be taken to observe subtleties in methods of preparing traditional medicines that could produce similar effects.

Aside from the gut microbiome, the human small intestine is one of the most significant sites for the secretion of β-glucosidases, making it the most important site for the absorption of flavonoid aglycones (Németh et al., 2003). As previously stated, differences in bioactivity of compounds in their glycosidic Vs aglycone forms challenges the reproducibility of antimicrobial or immunomodulatory outcomes.

The role for essential oils as anti-infective agents is another of the problematic ethnopharmacological research areas. Antimicrobial outcomes are naively extrapolated to pathologies that require dangerously high systemic concentrations to achieve contact inhibition. Generally, antimicrobial outcomes with essential oils are expected to have implications mainly for topical therapies, because MIC concentrations are not realistically achieved systemically. However, essential oils confer immunomodulatory effects at substantially lower concentrations.

Positive therapeutic outcomes may be a possibility, but a more detailed examination of mechanism of action is necessary. In the 1960s a sweetener called safrole was added to beverages. It was subsequently demonstrated to lead to hepatotoxicity and cancers in mice, so its use was discontinued. Although no significant antimicrobial activity has been attributed to the preconjugated form of safrole, a role in immunomodulation has been identified (Sa et al., 2014). Many essential oils, not just phenylpropanoids, have demonstrated immunomodulatory effects (Anastasiou and Buchbauer, 2017) emphasizing again that antimicrobial assays *per se* may not wholly explain the presumed therapeutic efficacy of an aromatic medicinal plant.

Essential oil metabolites may have lower MIC values or may even be toxic. As previously mentioned, it is difficult to predict how the conjugated xenobiotic will look, but insight can be garnished from *in vivo* pharmacokinetic studies. For example, studies on safrole demonstrated that the carcinogenic compound was not actually safrole, but the phase-1 metabolites 1’-hydroxysafrole and 1’-hydroxy-2’,3’-oxide. In addition, phase-2 metabolites 1’-acetoxysafrole and safrole-1’-sulfate, were shown to be mutagenic (Wislocki et al., 1997). These metabolites (Figure 9) were identified by detailed study of the urine of mice.

Knowledge of the metabolite form of sulfate esters of coumarins is another research area that could contribute to our understanding of the immunomodulatory activity of many species. Most notably, not much is known of the sulfate esters of coumarins in *Pelargonium sidoides* (Hauer et al., 2010), a South African medicinal root that is now marketed out of Germany under the name ‘Umckaloabo’. Its main therapeutic claim is for coughs and colds, but extracts show low activity upon screening for antimicrobial compounds against respiratory pathogens. In this case, the putative active compounds are possibly created during metabolism. Alternatively, anti-infective activity may be partly or wholly explained by immunomodulatory effects, but this requires further investigation.

The commercial success of *Pelargonium sidoides* as a treatment for respiratory afflictions under the name of Umckaloabo (EPs^®^ 7630) is due to the efforts of Charles Henry Stevens (Brendler and Van Wyk, 2008), who in 1897 traveled from England to South Africa upon recommendation from his doctor to experience relief from tuberculosis. It was believed at the time that the clean air was the point of difference that accommodated recovery from his affliction. After consultation with a Lesotho sangoma he was prescribed a remedy that greatly accelerated his recovery, or so the legend goes. As previously mentioned, today the mechanism of this remedy has eluded researchers, but some indication of immunomodulation is evident *in vitro* (Brendler, 2009).

## Vitamin D

While it is conceivable that ‘clean air’ may have played a role in Steven’s recovery from tuberculosis, recent studies indicate that the ‘African sun’ is more likely to have been an important complement to the efficacy of Umckaloabo. Vitamin D deficiency has been correlated with incidences of infection, particularly tuberculosis, and it is believed that supplementation or sunlight exposure (leading to Vitamin D UV-synthesis) can promote recovery (Gombart, 2009). However, using oral doses, clinical trials have not demonstrated consistent outcomes (Gombart, 2009) which may be related to insufficient dose or variable oral bioavailability (Alsaqr et al., 2015). The transdermal route is one proposed solution, but higher oral dose can also be useful, using rich natural sources, such as the Australian food species *Tasmannia lanceolata* (Poir.) A.C. Smith (Winteraceae) or *Solanum centrale* J.M. Black (desert resin: Solanaceae) (Black et al., 2017).

**FIGURE 10:**
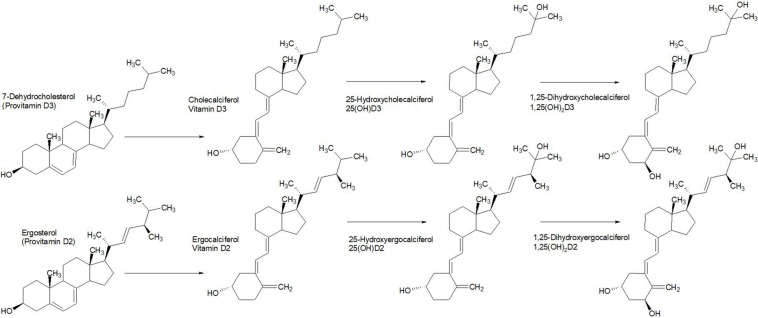
Biosynthetic precursors to Vitamin D3 (top) and D2 (bottom). Vitamin D3 and D2 differ by their alkyl substituent branching from the 5 membered ring. This difference is also evident in the 1,25-dihydroxy derivatives, which are the active immunomodulatory forms. No research has yet elucidated salient differences in biological functions.

The immunomodulatory effects of Vitamin D_3_ (from sunlight) and Vitamin D_2_ (from dietary sources) starts with the phase-1 liver metabolite 25(OH)D (Figure 10), which is converted to its active form 1,25(OH)_2_D by the mitochondrial 1 α-hydroxylase enzyme, the majority of which occurs in the primary renal tubules of the kidney (Gombart, 2009). It is postulated that 1,25(OH)_2_D regulates specific genes encoding for antimicrobial peptides. To date no studies have demonstrated a bioactivity difference between D_2_ and D_3_ forms of 1,25(OH)_2_D or the effects of using transdermal routes of precursors to by-pass ‘first-pass’ metabolism, which is inevitable in oral routes, and hence increase the half-life of its pre-conjugated form. Furthermore, no studies have explained or nullified the potential superiority of UV-synthesized routes of Vitamin D.

## Bioavailability Estimation in Practice: Worked Examples

**FIGURE 11:**
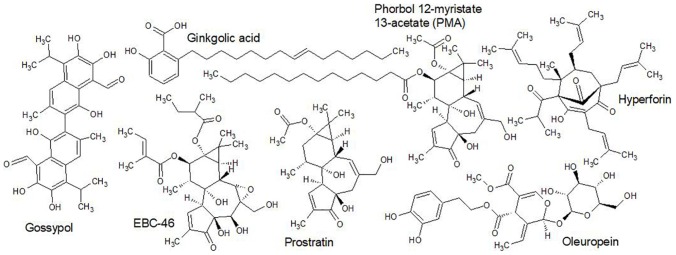
Structures with antimicrobial and immunomodulatory effects that illustrate important structural caveats related to bioavailability.

Another ‘nitrogen deficient’ class of compound that confers noteworthy antimicrobial activity, comparable to the chalcones and prenylated isoflavones, is the acylphloroglucinol, such as the synthetic PPAP 23 (MIC 1 μg/mL) (Wang et al., 2019), or the naturally occurring hyperforin (Figure 11) from St John’s Wort (*Hypericum perforatum* L. Hypericaceae) (Lyles et al., 2017), also with an MIC value at 1 μg/mL against *Staphylococcus aureus* (Reichling et al., 2001). Although today St John’s Wort is commonly prescribed for psychological ailments, it was once prized for topical anti-infective effects. Its efficacy was reinforced by a doctrine of signatures comparison to human skin;

“The little holes where of the leaves of Saint John’s wort are full, doe resemble all the pores of the skin and therefore it is profitable for all hurts and wounds that can happen thereunto.” Coles, William (1657). Adam in Eden, or, Natures paradise. OCLC 217197164

An examination of the structure of hyperforin (Figure 11) using Veber’s descriptors (Veber et al., 2002) demonstrates that the polar headspace of 71.44 Å^2^ is nearly half of the prescribed cut off for bioavailability (140 Å^2^). Thus, its presence in the oil extract used in Kosovar traditional medicine (Lyles et al., 2017) is consistent with this observation, since low polar headspace values correlate to increased lipophilicity. The estimation of rotatable bonds, using the current definition, gives 11, which is above the cut off at 10 prescribed by Veber et al. (2002). However, some ambiguity may be experienced with single bonds to sp2 hybridized orbitals (single bond to a double bonded carbon). This is exemplified by examination of the 5,6-trans bond (*E*) in vitamin D and derivatives (Figure 10) where free rotation about the single bond replaces *trans* with *cis* bonds, which evidently doesn’t happen without energy input. Thus, rotation about a single bond between two sp2 hybridized carbons does not happen. This is slightly different to hyperforin however, since the single bond is between a methylene and a singular sp2 orbital (not between two sp2 hybridized carbons). Nevertheless, exclusion of these bonds from the count of ‘free’ rotatable bonds lowers the total to 6, which may have significant implications for the interpretation of the bioavailability of this structure. In oral bioavailability studies maximum plasma levels were reached in 3 h (Biber et al., 1998), indicating good absorption, an apparent contradiction of the bioavailability estimation, if the definition of rotatable bonds is not tightened.

Another area of ambiguity is on structures with alkyl or fatty acid ester side chains. Chains longer than 10 carbons have 10 or more rotatable bonds, theoretically but not actually reducing bioavailability. In reality, alkyl chains are considered to enhance bioavailability by conferring lipophilicity to one side of the molecule. Lengths in the range of 10–15 carbons often optimize for antimicrobial efficacy against Gram-positive organisms. Ginkgolic acid from leaves and seeds of *Ginkgo biloba* L. (Ginkgoaceae) is a good example of this (Hua et al., 2017), with 14 rotatable bonds and polar head space of 57.53 Å^2^ (Figure 11). But due to poor aqueous solubility, it is unclear how suitable topically applied ginkgolic acid would be without adequate formulation. After ingestion by mice, plasma concentrations were measured, confirming oral bioavailability. With rapid metabolism and return to the bowel, ginkgolic acid is eliminated almost exclusively in feces (Xia et al., 2013). Nevertheless, it is advisable to exercise caution when interpreting numbers of rotatable bonds where homologous series of methylene carbons are present (repeating CH_2_ units; i.e., -CH_2_-CH_2_-CH_2_- and so on), such as with alkyl or fatty acid ester groups.

Fatty acid esters in phorbols can increase toxicity by enhancing penetration into phospholipid membranes, the site where the drug’s mechanisms are enacted (Goel et al., 2007). Phorbol esters belong to a class that is reputably either toxic or in a dramatic twist, significantly therapeutic. They are best known for tumor promotion by activation of protein kinase c (PKC). The standard tumor promoter that is used as a positive control in toxicity studies is phorbol 12-myristate-13-acetate (PMA), which as the name suggests, has a fatty acid ester, substantially increasing hydrophobicity (Boyle et al., 2014). The number of rotatable bonds is 17, or 4 if the myristate moiety is merely counted as one and polar headspace is 130.36 Å^2^ (Figure 11). Evidently this highly toxic bioavailable phorbol ester breaks the rules set out by Veber et al. (2002) if it is not recognized that the number of rotatable bonds in the fatty chain complicates the process of bioavailability estimation.

It is ironic that in the same class of compound as PMA, one of the most potent anticancer drugs are found, which is now in phase-2 human clinical trials. Tigilano tiglate (EBC-46; Figure 11) was isolated from the Australian rainforest species *Fontainea picrosperma* C.T.White (Euphorbiaceae). This drug also regulates PKC expression, but it activates a more specific subset of isoforms compared to the previously mentioned PMA (Boyle et al., 2014). With 8 rotatable bonds and polar headspace of 159.82 Å^2^ this drug is slightly more hydrophilic than is acceptable by the guidelines proposed by Veber et al. (2002). However, this drug is normally administered by injection directly into the tumor mass (intratumoral).

When the immunomodulatory Polynesian drug prostratin (Figure 11), a phorbol ester, was first isolated from a medicinal species in Samoa (*Homalanthus nutans* (G.Forst.) Guill, Euphorbiaceae), it was immediately assumed it would be dangerous in human use, but *in vitro* studies demonstrated its safety and further identified HIV activation of latently infected CD4^+^ T cells and exposing them to immune response, which reduces the pathogenicity of the HIV virus (Beans et al., 2013). Although prostratin is normally given by infusion, with a polar headspace of 139.59 Å^2^ and only 3 rotatable bonds, it is a good candidate for the transdermal or oral route, such as in the traditional Samoan practice. Indeed, a concept for a slow release oral tablet has been proposed (Brown and Hezarah, 2012).

Another HIV inhibitory compound, also with antimalarial and antibacterial properties, is the polyphenol gossypol, which is isolated from the cotton plant (*Gossypium hirsutum* Malvaceae) (Polsky et al., 1989). This drug is a dimer of heptyl-substituted naphthalene, with aldehyde and OH substituents (Figure 11). It is worthy of mention because of the unconventional chiral center as the bridge of the dimer, which is a single C-C bond. Due to the OH substitution of the aromatic carbons adjacent to the single bond free rotation is prevented, causing two enantiomeric forms to exist (Keshmiri-Neghab and Goliaei, 2014). Generally, the negative enantiomer is most cited in association with bioactivities. Since the bridging bond is not freely rotatable, the structure has only 4 rotatable bonds and a slightly high polar headspace of 155.52 Å^2^, which may slow the rate of absorption of this compound.

The final example is of oleuropein (Figure 11), an immunomodulatory seco-iridoid with a sugar moiety that is most famously derived from aerial parts of the olive tree (*Olea europaea* L.) (Vezza et al., 2017). Not only is this drug able to confer anti-inflammatory effects in the tissues of the intestines, but it also modifies the immune response in a positive way by increasing IFN-y production, which is associated with higher absolute numbers of CD8 + and NK (natural killer) cells (Magrone et al., 2018). Oleuropein has 10 freely rotatable bonds and a polar headspace of 201.67 Å^2^ it is unlikely to be absorbed passively in the human intestine. However, active transport of monoglycosides occurs on the Na^+^/glucose cotransporter.

Thus, while there are many valuable natural products that break rules related to passive bioavailability, exceptions can often be made where factors related to active transport mechanisms, alkyl side chains, rotational energy barriers or optimal steric placement of functional groups can influence bioavailability. Such factors need to be given careful consideration when bioavailability estimation is attempted.

## Conclusion

The widespread emergence of common pathogens resistant to frontline antibiotics has prompted an increasingly desperate search not just for new ‘magic bullets’ but also for new strategies to deploy and administer existing drugs. Plant secondary metabolites provide a potential treasure trove in this regard. In this review we have surveyed a range of pertinent investigations from our own and other laboratories. By introducing a number of important caveats, we warn against naive extrapolation from *in vitro* laboratory results to therapies that may be available to clinicians at some future date. Our aim is not to discourage the very valuable work currently being undertaken, particularly in the ethnopharmacological domain, but rather to provide indications based on relatively simple metabolic and chemical principles that may sharpen and concentrate the focus of researchers in the field.

## Author Contributions

NS wrote the manuscript. GJ assisted with edits.

## Conflict of Interest

The authors declare that the research was conducted in the absence of any commercial or financial relationships that could be construed as a potential conflict of interest.
